# Phylogenetic Relationships Within the Hyper-Diverse Genus *Eugenia* (Myrtaceae: Myrteae) Based on Target Enrichment Sequencing

**DOI:** 10.3389/fpls.2021.759460

**Published:** 2022-02-04

**Authors:** Augusto Giaretta, Bruce Murphy, Olivier Maurin, Fiorella F. Mazine, Paulo Sano, Eve Lucas

**Affiliations:** ^1^Faculdade de Ciências Biológicas e Ambientais, Universidade Federal da Grande Dourados, Unidade II, Dourados, Brazil; ^2^Laboratório de Sistemática Vegetal, Departamento de Botânica, Instituto de Biociências, Universidade de São Paulo, São Paulo, Brazil; ^3^Jodrell Laboratory, Royal Botanic Gardens, Kew, Surrey, United Kingdom; ^4^Department of Life Sciences, Imperial College, London, United Kingdom; ^5^Centro de Ciências e Tecnologias para a Sustentabilidade, Universidade Federal de São Carlos, Campus Sorocaba, Sorocaba, Brazil; ^6^Herbarium, Royal Botanic Gardens, Kew, Surrey, United Kingdom

**Keywords:** high-throughput sequencing, Hyb-Seq, target sequence capture, phylogenomics, Myrtaceae, *Eugenia*

## Abstract

*Eugenia* is one of the most taxonomically challenging lineages of flowering plants, in which morphological delimitation has changed over the last few years resulting from recent phylogenetic study based on molecular data. Efforts, until now, have been limited to Sanger sequencing of mostly plastid markers. These phylogenetic studies indicate 11 clades formalized as infrageneric groups. However, relationships among these clades are poorly supported at key nodes and inconsistent between studies, particularly along the backbone and within *Eugenia* sect. *Umbellatae* encompasses ca. 700 species. To resolve and better understand systematic discordance, 54 *Eugenia* taxa were subjected to phylogenomic Hyb-Seq using 353 low-copy nuclear genes. Twenty species trees based on coding and non-coding loci of nuclear and plastid datasets were recovered using coalescent and concatenated approaches. Concordant and conflicting topologies were assessed by comparing tree landscapes, topology tests, and gene and site concordance factors. The topologies are similar except between nuclear and plastid datasets. The coalescent trees better accommodate disparity in the intron dataset, which contains more parsimony informative sites, while concatenated trees recover more conservative topologies, as they have narrower distribution in the tree landscape. This suggests that highly supported phylogenetic relationships determined in previous studies do not necessarily indicate overwhelming concordant signal. Congruence must be interpreted carefully especially in concatenated datasets. Despite this, the congruence between the multi-species coalescent (MSC) approach and concatenated tree topologies found here is notable. Our analysis does not support *Eugenia* subg. *Pseudeugenia* or sect. *Pilothecium*, as currently circumscribed, suggesting necessary taxonomic reassessment. Five clades are further discussed within *Eugenia* sect. *Umbellatae* progress toward its division into workable clades. While targeted sequencing provides a massive quantity of data that improves phylogenetic resolution in *Eugenia*, uncertainty still remains in *Eugenia* sect. *Umbellatae*. The general pattern of higher site coefficient factor (CF) than gene CF in the backbone of *Eugenia* suggests stochastic error from limited signal. Tree landscapes in combination with concordance factor scores, as implemented here, provide a comprehensive approach that incorporates several phylogenetic hypotheses. We believe the protocols employed here will be of use for future investigations on the evolutionary history of Myrtaceae.

## Introduction

*Eugenia* P. Micheli ex L. is arguably one of the most taxonomically challenging lineages of flowering plants, with more than 1,100 species distributed throughout tropical and subtropical areas but with a main center of diversity in the Neotropics (Wilson, 2011; World Checklist of Selected Plant Families [WCSP], 2021). *Eugenia* is a dominant tree genus in some tropical forest biomes, has high ecological importance for frugivores, and is used as a surrogate for tree species diversity and evolution in Neotropical biomes (Oliveira-Filho and Fontes, 2000; Lucas and Bünger, 2015). For many years, *Eugenia* existed as a morphologically homogeneous genus with a well-delimited circumscription (Candolle, 1828; Berg, 1857). Later, the understanding of *Eugenia* was enhanced by recognition and description of its growing species diversity especially in the Neotropics (e.g., Berg, 1857; Amshoff, 1951; McVaugh, 1956; Landrum and Kawasaki, 1997). Biological classifications are permanently “works in progress” (e.g., The Angiosperm Phylogeny Group [APG], 1998, 2003, 2009, 2016), and this is particularly true for *Eugenia*, where morphological delimitation has substantially changed over the last few years. These changes have been driven by recent phylogenetic studies based on molecular data (van der Merwe et al., 2005; Cruz et al., 2013; Mazine et al., 2014; Bünger et al., 2016a; Mazine et al., 2018; Giaretta et al., 2019b; Flickinger et al., 2020). According to these molecular phylogenetic reconstructions, the following formerly segregated genera are now treated under *Eugenia*: *Calycorectes* O.Berg, *Catinga* Aubl., *Calyptrogenia* Burret, *Hexachlamys* O. Berg, *Hottea* Urb., *Jossinia* Comm. ex DC., *Phyllocalyx* O.Berg, *Pseudanamomis* Kausel, and *Stenocalyx* O.Berg (see World Checklist of Selected Plant Families [WCSP], 2021).

Information from several sources has changed morphological understanding and challenged generic concepts in *Eugenia*. For example, the standard tetramerous flower in *Eugenia* is nearly ubiquitous except in *Hexachlamys* (currently treated under *Eugenia*), which can express penta- and hexamerous flowers (Cruz et al., 2013; Vasconcelos et al., 2018). Flowers with six apparent petals, previously placed in *Calycorectes*, also appear to depart from the basic arrangement in *Eugenia*, although a detailed study on flower development, in fact, reveals a pair of internal sepals expressing petal-like features (Giaretta et al., 2019a,b). Furthermore, species with a “six petals” display are associated with fused calyces, a condition contrasting with free lobes found in the basic arrangement of the genus (Landrum and Kawasaki, 1997). Likewise, species previously assigned to *Phyllocalyx* because of remarkably extended free sepals (Berg, 1857) that enlarge quickly in early development stages (Vasconcelos et al., 2018) are currently treated as an infrageneric group supported by morphological and molecular data (Bünger et al., 2016a; Mazine et al., 2016; Mazine et al., 2018).

**FIGURE 1:**
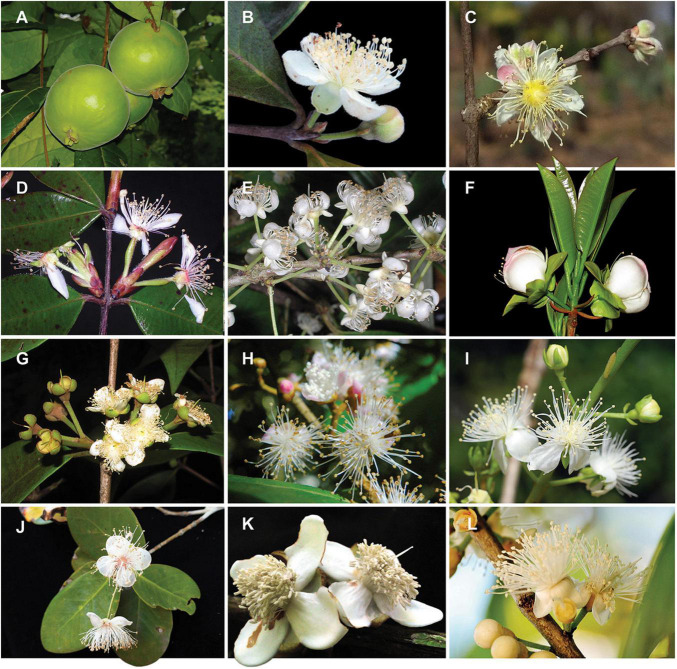
Diversity of infrageneric groups of *Eugenia.* **(A)** *E. stipitata* – sect. *Pilothecium.* **(B)***E. klotzschiana* – sect. *Pseudeugenia.* **(C)** *E. myrcianthes* – sect. *Hexachlamys.* **(D)** *E. neosilvestris* – sect. *Eugenia.* **(E)** *E. excelsa* – sect. *Excelsae.* **(F)** *E. involucrata* – sect. *Phyllocalyx.* **(G)** *E. acutata* – sect. *Schizocalomyrtus.* **(H)** *E. disperma* – sect. *Racemosae.* **(I)** *E. bunchosiifolia* – sect. *Speciosae.* **(J)** *E. reinwardtiana* – sect. *Jossinia.* **(K)** *E. abunan* – sect. *Umbellatae.* **(L)** *E. pluriflora* – sect. *Umbellatae.* Size of reproductive organs varies between c. 1 and 7 cm. Pictures by *A. Giaretta* **(A,D,E–G)**, *K. Valdemarin* **(B)**, *P. Gaem* **(C)**, *A. Maruyama* **(H)**, *E. Lucas* **(I)**, Y.W. Low **(J)**, M. Simon **(K)**, O.J. Pereira **(L)**.

Phylogenetic reconstructions based on Sanger sequencing resulted in the classification of *Eugenia* comprising 11 infrageneric groups (Figure 1). These studies used both plastid and nuclear markers with a multitude of plastid markers, while the nuclear genome was often represented only by the ITS region (e.g., Mazine et al., 2018; Giaretta et al., 2019b; Flickinger et al., 2020). In practice, this means resulting phylogenetic trees are largely reconstructions of the genealogical history of the plastid genome rather than consensus between this and the nuclear genome. The implication for *Eugenia* systematics remains unclear, because the few commonly used nuclear markers are not sufficient to reliably tackle genealogical comparative topologies. Despite this, phylogenies inferred from concatenation of both genomes converged on strongly monophyletic *Eugenia* and foundation for the infrageneric classification of 11 the groups (Mazine et al., 2016; Mazine et al., 2018). However, statistical support for relationships among the infrageneric groups remains low especially in the backbone of *Eugenia*. Coalescent-based methods, which, in theory, can provide a more reliable species tree when individual markers are in conflict, have not been used in a comprehensive approach. Therefore, uncertainty accompanies the taxonomic classification in which *Eugenia* is currently divided into subgenera *Eugenia*, *Hexachlamys*, and *Pseudeugenia*. Subgenus *Eugenia* encompasses the great majority of species, and includes nine sections, while the subgenera *Hexachlamys* and *Pseudeugenia* include one homonymous section each (Mazine et al., 2018). Efforts to reconcile recent molecular phylogenies with morphological features have resulted in the current taxonomic arrangement in *Eugenia* (Mazine et al., 2016; Giaretta et al., 2019b). Nevertheless, the highly diverse *Eugenia* sect. *Umbellatae* O.Berg, which includes ca. 700 species, has low support at internal nodes and remains the major challenge. Furthermore, despite a preliminary survey (Mazine et al., 2018), morphological synapomorphies have not been identified to distinguish internal divisions of this section. These issues have impeded the development of a reliable classification and continue to render *Eugenia* sect. *Umbellatae* taxonomically unmanageable (Mazine et al., 2018). To further clarify phylogenetic relationships and understand the evolution of morphological traits within *Eugenia*, a methodological step-change that will add significantly more molecular data than previous Sanger sequencing efforts is desirable.

In this study, we aim to reconstruct relationships within *Eugenia* using the universal Angiosperm-353 target sequencing kit, which uses a probe set designed to selectively capture low-copy nuclear orthologs (Johnson et al., 2019). This approach generates comprehensive phylogenies entirely based on the nuclear genome, with additional recovery of off-target plastid sequences also possible; this is the first use of phylogenomic methods in *Eugenia.* Specific objectives of this study are to: (1) test the concordance of phylogenetic reconstructions based on nuclear and plastid data; (2) assess the effectiveness of increasing genetic data from diverse sources (intron and intergenic spacers) to increase statistical support for relationships; (3) compare topologies from phylogenetic reconstructions based on concatenated and multi-species coalescent (MSC) approaches; and (4) establish well-supported systematic relationships in *Eugenia*, particularly within *Eugenia* sect. *Umbellatae*.

## Materials and Methods

### Molecular Sampling

Material composed of 54 taxa was successfully sequenced from the DNA (32 samples) and dried leaf tissue (22 samples) banks at the Royal Botanic Gardens, Kew (RBG-Kew). The sampling strategy adopted was to include as many species as possible, representing all previously recognized infrageneric groups, to resolve relationships along the *Eugenia* backbone and within *Eugenia* sect. *Umbellatae*. The only group not represented, because of unsuccessful recovery, was *Eugenia* sect. *Hexachlamys*. Seven outgroup taxa were used, such as *Myrcianthes*, which is shown as sister to *Eugenia* in previous molecular phylogenies based on Sanger sequencing (e.g., Mazine et al., 2014; Giaretta et al., 2019b). Outgroup sequence data are taken from the Myrtales-focused study of Maurin et al. (2021) and are available on the Kew Tree of Life Explorer (https://treeoflife.kew.org; Baker et al., 2021). Sample voucher details are provided in Supplementary Table 1.

### DNA Extraction and Library Preparation

Total DNA was extracted from each of the 22 silica-dried leaf samples with QIAGEN^®^ *DNeasy*^®^ Plant Maxi Kits, using 0.2 g of the ground tissue material to generate 1.5 ml of solution, as per the manufacturer’s protocol. Fragment size was estimated using Agilent 4200 TapeStation (Agilent Technologies, Palo Alto, CA, United States). To homogenize median fragment size across all samples, those with fragment sizes >400 bp were sonicated in Covaris AFA Fiber Pre-Slit Snap-Cap micro TUBEs for 40 s using Covaris E220 Focused-Ultrasonicator (Covaris, Inc., Woburn, MA, United States) with peak power set to 50W and duty factor at 20%.

Sequencing libraries were created with the NEBNext Ultra (New England Biolabs, Ipswich, MA, United States) kit, using 200 ng of fragmented DNA for each sample and size-selecting at 550 bp with Ampure magnetic beads. The libraries were prepared in 25 μl (half volume) to maximize reagents and indexes, and then amplified through eight cycles of PCR amplification. DNA was quantified using a Quantus (Promega Corporation, Madison, WI, United States) fluorometer, and fragment size was estimated using TapeStation. Dual indexed samples were pooled with up to 50 ng of library DNA for enrichment using an Angiosperm-353 v1 target capture kit (Johnson et al., 2019) available from Arbor Biosciences (Ann Arbor, MI, United States), performed for over 24 h at 65°C. Enriched products were PCR-amplified for 12 cycles and sequenced on Illumina MiSeq (Illumina, San Diego, CA, United States) with v3 reagent chemistry (2 × 300-bp paired-end reads) at the Royal Botanic Gardens (Kew, United Kingdom).

### Data Processing and Recovery of Target Loci

Raw sequences were quality-filtered, and adapters were removed using Trimmomatic (Bolger et al., 2014) in Illumina clip palindrome mode following the settings available in Murphy et al. (2020). The resulting cleaned data were used to assemble loci *via* the *HybPiper* pipeline v1.3.1 (Johnson et al., 2016) using the BLAST option. Orthologous nuclear coding sequences (CDS) and the non-coding “splash zone” encompassing intron and intergenic DNA fragments flanking the target exons (INT) were both targeted for recovery for each of the 353 loci in the multi-species amino acid target file (Johnson et al., 2019). Additionally, off-target plastid reads were recovered using a target file composed of 78 *Eugenia uniflora* genes (excluding duplicates) downloaded from GenBank (accession number NC_027744.1; Eguiluz et al., 2017). The coverage cut-off option was set to four for both recoveries. *HybPiper* indicates loci with potential paralogs (Johnson et al., 2019). These were excluded when recurrent in more than three samples, which was the case only for the nuclear loci: g4527 (gene PTAC10), g5578 (PSAG), g5910 (HCF136), g6003 (LPA1), and g6376 (GCP4). For more information on the genes, see https://treeoflife.kew.org/gene-viewer.

### Datasets and Alignment

Sampling was based on nuclear and plastid databases encompassing *Eugenia* and its tribe, Eugeniinae, as well as six outgroups of other Neotropical Myrtaceae genera. CDS and INT were recovered from the nuclear dataset, while only CDS was used from the plastome. Ambiguous alignment and low phylogenetic signal prevented the use of plastid intronic sequences. This resulted in three nuclear datasets [coding (ncCDS), introns (ncINT), a combination called genomic dataset (ncGD)], and one plastid dataset (see Table 1 for acronyms). The plastid dataset (plCDS) only included *Myrcianthes* as outgroup to avoid ambiguous alignment. Multiple species alignments were performed using the default settings of PASTA v1.8.5 (Mirarab et al., 2015). Spurious alignment sites and gaps were removed using the “-gt 0.2” option of trimAl v1.3 (Capella-Gutiérrez et al., 2009), which removes all sites with gaps in more than 80% of the sequences.

**TABLE 1:** Acronyms for datasets and trees.

Datasets	
ncCDS	Nuclear orthologous sequences of coding regions
ncINT	Nuclear splash zone which encompass introns and intergenic DNA that flank target exons
ncGD	Combination of ncCDS and ncINT
plCDS	Plastid orthologous sequences of coding regions

**Trees**	

As	Coalescent Astral tree with local posterior probability support
Abs	Coalescent Astral tree with 100 bootstrap replicates for branch support
AUFbs	Coalescent Astral tree with 1,000 ultrafast bootstrap replicates for branch support
Cpa	Concatenated and partitioned ML reconstruction with bootstrap support
Cun	Concatenated and unpartitioned ML reconstruction with bootstrap support

### Model Violation and Loci Selection

Model violation of partition sequence evolution was tested (Naser-Khdour et al., 2019). Loci that did not fit the test of symmetry implemented in IQ-TREE 2.0 (Nguyen et al., 2015; Minh et al., 2020) through the command “–symtest-remove-bad” using default parameters (*p*-value = 0.05) were excluded before evolutionary model calculation and phylogenetic reconstruction (Supplementary Table 2). ModelFinder (Kalyaanamoorthy et al., 2017) was then used to determine the best substitution models of molecular evolution according to the Bayesian Information Criterion (BIC) implemented in IQ-TREE.

### Phylogenetic Reconstruction

Concatenation and multispecies coalescent model (MSC)-based approaches were implemented for the phylogenetic reconstruction (see Table 1 for acronyms). The four datasets (ncCDS, ncINT, ncGD, and plCDS) were independently concatenated into four matrices with AMAS.py (Borowiec, 2016) and used as input to a fully partitioned scheme that estimates best-fit substitution models by loci, and an alternative unpartitioned scheme (also referred to as “supergene,” e.g., Gonçalves et al., 2019) that assumes only one substitution model. This resulted in eight concatenated species trees. Tree recovery was performed using maximum likelihood (ML) reconstruction in IQ-TREE. The option “-spp” was used in the concatenated fully partitioned scheme, allowing all partitions to share the same relative branch length while maintaining their own evolutionary model (Duchêne et al., 2019). Different metrics of branch support were compared. The parametric aBayes exhibits best power in scenarios of low or mild model violations and tends to give results very similar to the MCMC posterior probability (Anisimova et al., 2011). Additionally, more conservative branch support tests were performed using ultrafast bootstrap 2 (UFbs) (Hoang et al., 2018) and non-parametric SH-aLRT that calculates a unique branch support for the node instead of a summary of three confidence values, a robust approach for short branches (Guindon et al., 2010), implementing 1,000 replicates each. Both UFbs and SH-aLRT are believed to underestimate branch support. Additionally, individual locus alignments based on the ncCDS, ncINT, and plCDS datasets were used for gene tree inference in IQ-TREE using the same parameters as the concatenated full partition scheme except that only the UFbs was used for branch support. Resulting gene trees were used as input for the MSC approach using ASTRAL-III (Zhang et al., 2018), which uses a maximum quartet support method to produce species trees statistically consistent under the MSC model as long as gene trees are error-free (Mirarab et al., 2014; Mirarab and Warnow, 2015). We built three independent species trees with local posterior probability (-t 3) branch support values. Additionally, MSC trees using 1,000 UFbs replicates and 100 bootstrap (bs) replicates of each gene tree (for a total of 353 genes, it would be ca. 353,000 and ca. 35,300 input gene trees, respectively) were recovered for each dataset, resulting in six independent species trees. The gene trees from the ncCDS and ncINT datasets were used as input for the MSC approach in the genomic dataset (ncGD); 1,000 UFbs replicates and 100 bootstrap (bs) replicates were implemented per gene tree, resulting in three independent species trees. Thus, a total of 20 species trees were recovered, and their topologies were compared. Statistical branch support was considered high for local posterior probability and bootstrap values when ≥0.9 and ≥90, respectively, and low when <0.75 and <75, respectively.

### Assessment of Congruence

Assessment of the tree landscape was performed to investigate congruence among the 20 species trees, contrasting the concatenated method with the MSC model. The statistical distribution of tree topology distance was calculated using the Robinson-Foulds algorithm (Robinson and Foulds, 1981) for unrooted trees and the Kendall-Colijn algorithm (Kendall and Colijn, 2016) for rooted trees. The Robinson-Foulds (RF) metric is based on the distance between clades of the different unrooted trees. The Kendall-Colijn (KC) metric calculates distances between pairs of taxa and their most recent common ancestor, and from that node to the root. The R package treespace v.1.1.3.2 (Jombart et al., 2017) was used to explore the phylogenetic landscape and identify similar topologies through hierarchical clustering of principal components using the function “findGrove.” Outgroups and six more tips (*Eugenia nutans* O.Berg, *E. aubletiana* Mattos, *E. neograndifolia* Mattos, *E. fasciculiflora* O.Berg, *E. flavescens* DC., and *E. batingabranca* Sobral) were removed from the species trees using the function “drop.tip” of the package ape v.5.3 (Paradis et al., 2004) to assemble an input file containing species trees with the same tips, as required for treespace. The function “reroot” of the package phytools v.0.6-99 (Revell, 2012) was used to root the trees on *Myrcianthes fragrans*. Plots and graphs were generated using ggplot2 v.3.2.1.

Statistical significance of incongruence between topologies was assessed using a partitioned concatenated tree produced using ML (fixed tree) and compared against each species trees. The ML-partitioned concatenated tree was used in this step, because it is closest to the arrangement used in previous molecular phylogenetic reconstructions in *Eugenia* (e.g., Bünger et al., 2016a; Mazine et al., 2016; Mazine et al., 2018; Giaretta et al., 2019b). The target ncCDS was used to run the fixed tree. Shimodaira-Hasegawa (SH) and approximately unbiased (AU) (Shimodaira and Hasegawa, 1999; Shimodaira, 2002) topology tests were performed to contrast a fixed tree with a set of all species trees. For this analysis, 10,000 resampling estimated log-likelihood (RELL) replicates were used with the same set of species trees (with dropped tips) implemented for treespace. The SH test tends to be more conservative, increasing the probability of recovering a false positive (topologies are statistically similar), while AU accounts for reduction in less conservative probabilities (Shimodaira, 2002). To further investigate conflicting signal among loci and across sites, the gene concordance factor (gCF) and site concordance factor (sCF) (Minh et al., 2020) were calculated for each node using the options “–gcf” and “–scf 100” in IQ-TREE. The gene and site concordance factors (gCF and sCF) are expressed in percentages and intuitively express underlying variance in data supporting a branch at both gene-level and site-level, irrespective of the size of the sample (Minh et al., 2020). Essentially, a concordance factor captures the proportion of single-locus trees consistent with a particular branch using a framework tree as reference. Astral trees of ncCDS, ncINT, and plCDS were used for reference. The gCF and sCF are used in conjunction with measures of statistical support to better depict the underlying process of phylogenetic reconstruction. The minimum score for the sCF is 33%, because it is calculated according to three possible resolutions of a quartet recovered at a node, while gCF varies from 0 to 100% (Minh et al., 2020). Gene concordance factor scores are classified as low (<30%), moderate (30–80%), and high (≥80%). These scores can be interpreted as follows [see a detailed example in Lanfear (2018), http://www.robertlanfear.com/blog/files/concordance_factors.html, accessed on 14/10/2021]. Similar gCF and sCF scores would suggest that the only source of discordance in the single locus tree is the conflicting signal caused, for instance, by genuine incomplete lineage sorting. Alternatively, a much lower gCF than sCF could indicate that other processes are involved, for instance stochastic error caused by limited signal for individual genes. High gCF and low sCF might indicate considerable overall noise that is nonetheless insufficient to distort the strong agreement between genes. Concordance factor scores of 14 recurrent clades in the species trees were viewed as heatmaps using the package pheatmap v.1.0.12 implemented in R.

## Results

### Data Retrieval

Summary statistics of the nuclear dataset indicate recovery of 1,815,065 reads with an average of 33,000 reads per sample (range: 12,222–143,714). On average, 292 loci were recovered per sample (range: 226–335); 132–268 loci (200 loci on average) had at least 50% coverage (Supplementary Figure 1). Nuclear CDSs were retrieved for 332 loci as well as 328 intron loci. Five potential paralog loci, 21 exons, and 84 introns did not fit the test of symmetry and were excluded, leaving 306 ncCDS loci and 239 ncINT loci for downstream analyses. For plastome off-target CDS (plCDS), 67,199 reads were recovered with an average of 1,083 reads per sample (range: 138–6,086). On average, 11 loci were recovered per sample (range: 1–56), with a maximum of 55 loci with at least 50% coverage (nine loci on average). An amount of 44 loci were retrieved for plCDS that fit the symmetry test.

The trimmed alignment length of individual loci of the ncCDS was between 59 and 2,713 bp (590 on average) with a total length of 179,123 bp, while the ncINT was 243–47,434 bp (1,375 on average) with total length of 328,564 bp (Table 2). The individual loci of the plCDS account for 183–5,613 bp (1,116 bp on average) with a total length of 49,437 bp. Parsimony informative sites (PIS) increased with alignment length (Supplementary Figure 2), except for the plastid dataset that maintained low levels of variation even in long alignments (Table 2). ncCDS had 11.9% PIS, and this increased to 26.9% in ncINT, at the cost of more missing data.

**TABLE 2:** Length of trimmed aligned contigs and parsimony informative sites (PIS) for the three datasets used in downstream analyses.

	Individual loci length (bp)							
	Total length (bp)	PIS	AT%	GC%	Min	Max	Average	Missing data (%)
ncCDS	179,123	21,257 (11.9%)	0.535	0.465	59	2,713	590	16.1
ncINT	328,564	88,606 (26.9%)	0.623	0.377	243	47,434	1,375	34.6
plCDS	49,437	484 (1%)	0.631	0.369	183	5,613	1,116	77.5

### Phylogenetic Relationships

A total of 20 species trees resulted from the different datasets (see Supplementary Figure 3) detailed in the methods. Differences in the resulting topologies are relatively minor with the greatest divergences found between the nuclear and plastid datasets. Of the 10 sampled infrageneric groups of *Eugenia*, eight were recovered as monophyletic according to the nuclear dataset. *Eugenia* sects. *Eugenia*, *Phyllocalyx*, and *Umbellatae* were monophyletic in both the plastid and nuclear datasets. *Eugenia* sects. *Excelsae*, *Schizocalomyrtus*, *Jossinia*, and *Racemosae* were not recovered as monophyletic in plCDS, and *E.* sect. *Excelsae* was not monophyletic in the ncINT concatenated trees. *Eugenia* sects. *Pseudeugenia* and *Pilothecium* were not monophyletic in all the reconstructions. *Eugenia* sect. *Speciosae* was represented only by *E. bunchosiifolia*, so its monophyly could not be tested.

**FIGURE 2:**
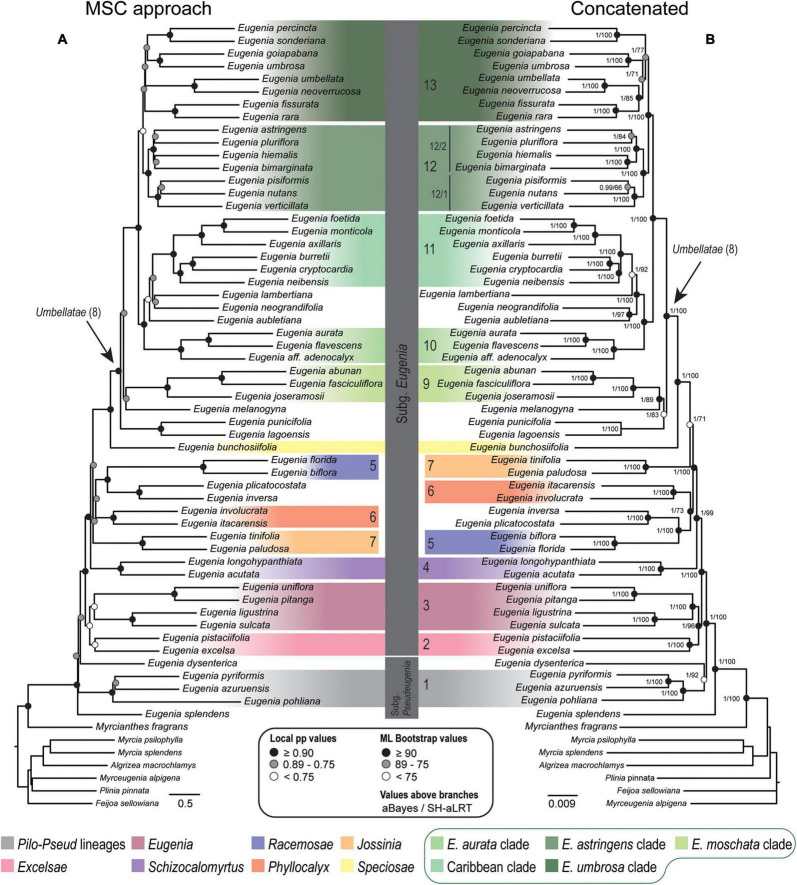
Phylogenetic reconstructions of *Eugenia* based on the 306 nuclear coding loci (ncCDS) targeted with the Angiosperm-353 probes. **(A)** Multi-species coalescent (MSC) tree using Astral with support values shown at nodes as local posterior probabilities (pp). **(B)** Concatenated partitioned tree with ML support shown at nodes as circles (see legend) and additional tests of branch support with values above branches (aBayes/SH-aLRT). Colored legend corresponds to the colored boxes (clades of *Eugenia* sect. *Umbellatae* are circled).

**FIGURE 3:**
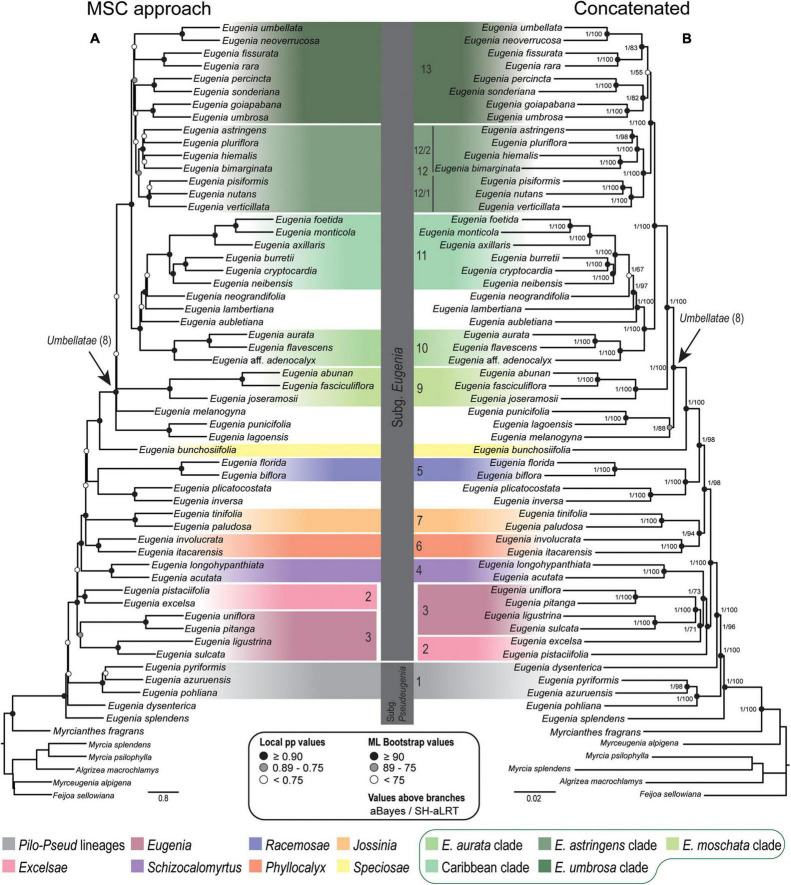
Phylogenetic reconstructions of *Eugenia* based on the 239 nuclear intron regions targeted with the Angiosperm-353 probes. **(A)** MSC tree using Astral with support values shown at nodes as local pp. **(B)** Concatenated partitioned tree with ML support shown at nodes as circles (see legend) and additional tests of branch support values above branches (aBayes/SH-aLRT). Colored legend corresponds to the colored boxes (clades of *Eugenia* sect. *Umbellatae* are circled).

**FIGURE 4:**
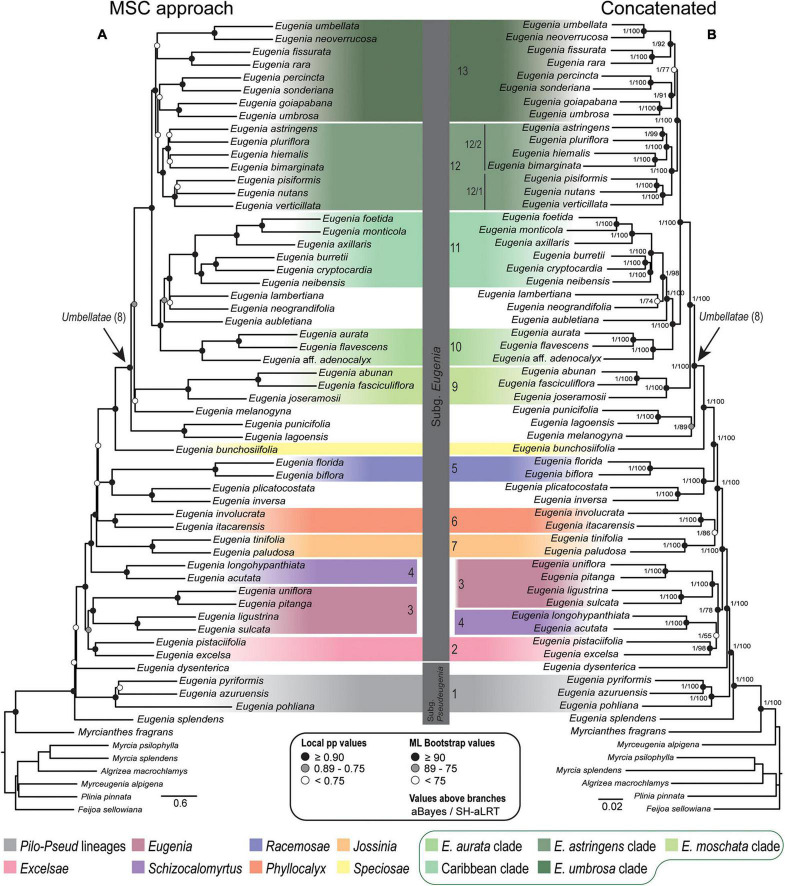
Phylogenetic reconstructions of *Eugenia* based on the 306 nuclear coding loci with 239 additional intron regions targeted with the Angiosperm-353 probes combined in a genomic dataset (ncGD). **(A)** MSC tree using Astral with support values shown at the nodes as local pp. **(B)** Concatenated partitioned tree with ML support shown at the nodes as circles (see legend) and additional tests of branch support with values above branches (aBayes/SH-aLRT). Colored legend corresponds to the colored boxes (clades of *Eugenia* sect. *Umbellatae* are circled).

Topologies from the ncCDS and ncINT (Figures 2, 3) datasets are similar, except for minor differences caused by uncertainty in the phylogenetic position of *Eugenia* sects. *Eugenia*, *Excelsae*, *Jossinia*, *Schizocalomyrtus*, *Phyllocalyx*, and *Racemosae*. Additionally, *Eugenia* sect. *Excelsae* was not recovered as monophyletic in concatenated ncINT. Improved support for these sections was returned in topologies from the ncGD dataset (Figure 4). Likewise, the ncGD dataset produced similar topologies, except for the position of *Eugenia* sect. *Eugenia* that is sister to sect. *Schizocalomyrtus* + *Excelsae* in the concatenated tree, in contrast to its placement as sister to sect. *Excelsae* in the MSC tree. Another source of uncertainty is the position of deeper clades in *Eugenia* sect. *Umbellatae*, where support is lower in all the three datasets.

### Tree Landscapes

**FIGURE 5:**
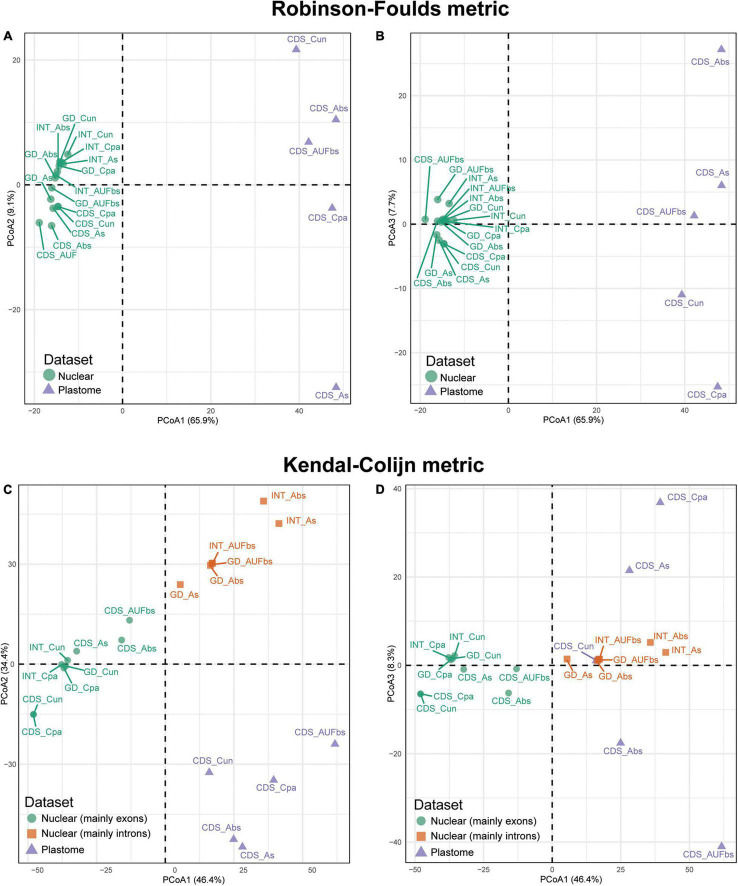
Principal coordinate analysis depicting ordinations of 20 tree topologies of Eugenia using Robinson-Foulds distance metric calculated from rooted trees **(A,B)** and Kendal-Colijn distance metric inferred from unrooted trees **(C,D)**. CDS, orthologous sequences of coding regions; INT, splash zone encompassing introns and intergenic DNA that flank target exons; GD, combination of nuclear ncCDS and ncINT; Cpa, concatenated and partitioned ML reconstruction; Cun, concatenated and unpartitioned ML reconstruction; As, coalescent Astral tree with local posterior probability support; Abs, coalescent-based Astral tree with 100 bootstrap replicates; AUFbs, coalescent Astral tree with 1,000 ultrafast bootstrap replicates).

Hierarchical clustering of the species trees in the landscape resulted in distinct distributions mainly driven by the datasets. Robinson-Foulds (RF) recovered two groups that correspond to the dataset, while the hierarchical clustering in three groups observed in the Kendall-Colijn (KC) is consistent with the combination of the dataset and the method of reconstruction (Figure 5). This is clearer when the distribution of the species trees is evaluated considering the three most informative axes. The three axes of the RF algorithm based on unrooted species trees showed that those based on plastid are distantly related, although they were nested in the same group (Figures 5A,B). Conversely, trees based on nuclear sequences are closely distributed. However, a slight segregation between ncCDS and ncINT can be observed, especially when axes 1 and 2 are assessed (Figure 5A). Species trees based on the genomic dataset (ncGD) are arranged close with those based on ncINT except for the Astral tree without replicates (Figure 5A, “see GD_As”) that was more similar to the trees based on the exon dataset. The reconstruction method employed seems not to make a significant difference to the distribution of the trees in the RF analysis, while no pattern was observed for the distribution of trees based on the plastid dataset.

The KC algorithm based on rooted species trees showed tendency of trees based on the same dataset to cluster and, to a lesser extent, trees from the same reconstruction method also had this tendency (Figures 5C,D). This is particularly evident on axes 1 and 2 (Figure 5C) that show plastid dataset trees concentrated at the bottom of the plot (triangles), while the nuclear dataset trees are on top (circles and squares). Species trees based on ncINT and ncGD datasets from Astral analysis are grouped (squares) while ncCDS group together (circles), including the ncINT concatenated trees. The distribution assessed with axes 1 and 3 is less informative, even though it supports the arrangement formed by ncCDS dataset trees (Figure 5D). The plastid dataset trees lack a clear arrangement.

### Topology Assessment

Table 3 shows the degree to which the dataset arrangement and reconstruction method influenced resulting topologies based on the tree topology tests. The more conservative SH test recovered dissimilarity in tree topologies with the plastid dataset regardless of the reconstruction method. The less conservative AU test showed that all topologies based on the plastid (plCDS) and intron (ncINT) datasets were different in comparison to the fixed tree. Topologies based on ncCDS and reconstructed using Astral were different from the fixed tree, whereas those reconstructed by a concatenation arrangement were statistically similar. Topologies resulting from ncGD were distinct from the fixed tree except for the Astral tree reconstructed without replicates.

**TABLE 3:** Tree topology test contrasting a fixed tree in bold (concatenated partitioned reconstruction based on ncCDS) with all species trees in which *p*-value < 0.05 is rejected (−) as similar to the fixed tree, while *p*-value > 0.05 is accepted (+) as having a similar topology with the fixed tree.

Dataset	Tree	SH		AU	
ncCDS	As	0.599	+	0.027	−
ncCDS	Abs	0.554	+	0.016	−
ncCDS	AUFbs	0.530	+	0.006	−
**ncCDS**	**Cpa**	**0.972**	**+**	**0.649**	**+**
ncCDS	Cun	1	+	0.638	+
ncINT	As	0.418	+	0	−
ncINT	Abs	0.430	+	0.001	−
ncINT	AUFbs	0.557	+	0.022	−
ncINT	Cpa	0.237	+	0.005	−
ncINT	Cun	0.177	+	0.001	−
ncGD	As	0.835	+	0.272	+
ncGD	Abs	0.531	+	0.025	−
ncGD	AUFbs	0.509	+	0.016	−
ncGD	Cpa	0.388	+	0.003	−
ncGD	Cun	0.405	+	0.005	−
plCDS	As	0	−	0	−
plCDS	Abs	0	−	0	−
plCDS	AUFbs	0	−	0	−
plCDS	Cpa	0	−	0	−
plCDS	Cun	0	−	0.001	−

*ncCDS, nuclear orthologous sequences of coding regions; ncINT, nuclear splash zone encompassing introns and intergenic DNA-flanking target exons; ncGD, genomic data as result of the combination of ncCDS and ncINT datasets; plCDS, plastid orthologous sequences of coding regions; Cpa, concatenated and partitioned ML reconstruction; Cun, concatenated and unpartitioned ML reconstruction; As, coalescent Astral tree with local posterior probability support; Abs, coalescent-based Astral tree with 100 bootstrap replicates; AUFbs, coalescent Astral tree with 1,000 ultrafast bootstrap replicates.*

### Assessment of Congruence

**FIGURE 6:**
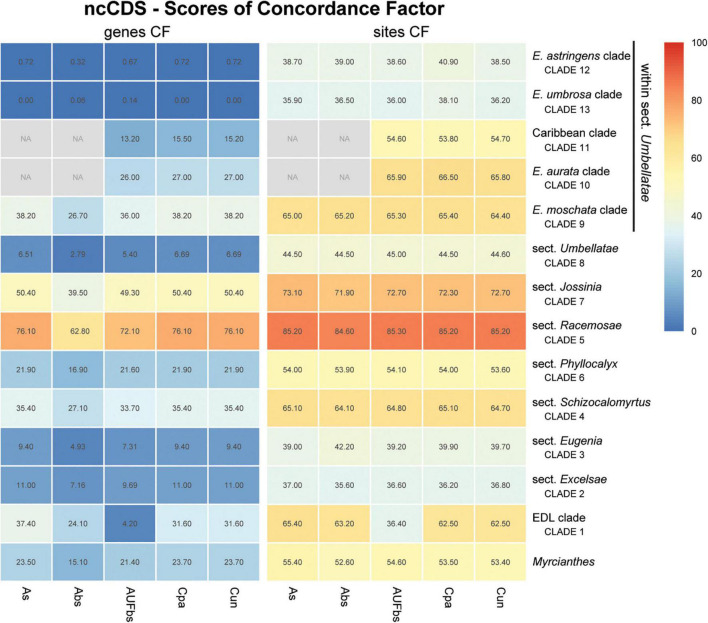
Heatmap of gene and site concordance factors (gCF and sCF) recovered in focus clades of the nuclear coding loci (ncCDS) in *Eugenia*. Each column is a method of phylogenetic reconstruction, and each row corresponds to a clade in Figures 2–4. Shading indicates percentage of concordance factor values recovered (Cpa, concatenated and partitioned ML reconstruction; Cun, concatenated and unpartitioned ML reconstruction; As, coalescent Astral tree with local posterior probability support; Abs, coalescent based Astral tree with 100 bootstrap replicates; AUFbs, coalescent Astral tree with 1,000 ultrafast bootstrap replicates; NA, node not recovered).

**FIGURE 7:**
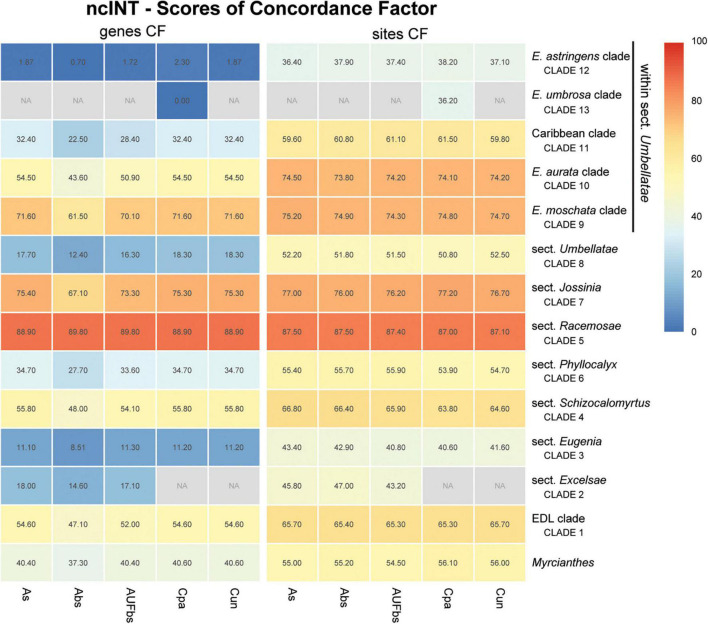
Heatmap of gene and site concordance factors (gCF and sCF) recovered in focus clades of the nuclear intronic non-coding loci (ncINT) in *Eugenia*. Each column is a method of phylogenetic reconstruction, and each row corresponds to a clade in Figures 2–4. Shading indicates percentage of concordance factor values recovered (Cpa, concatenated and partitioned ML reconstruction; Cun, concatenated and unpartitioned ML reconstruction; As, coalescent Astral tree with local posterior probability support; Abs, coalescent-based Astral tree with 100 bootstrap replicates; AUFbs, coalescent Astral tree with 1,000 ultrafast bootstrap replicates; NA, node not recovered).

**FIGURE 8:**
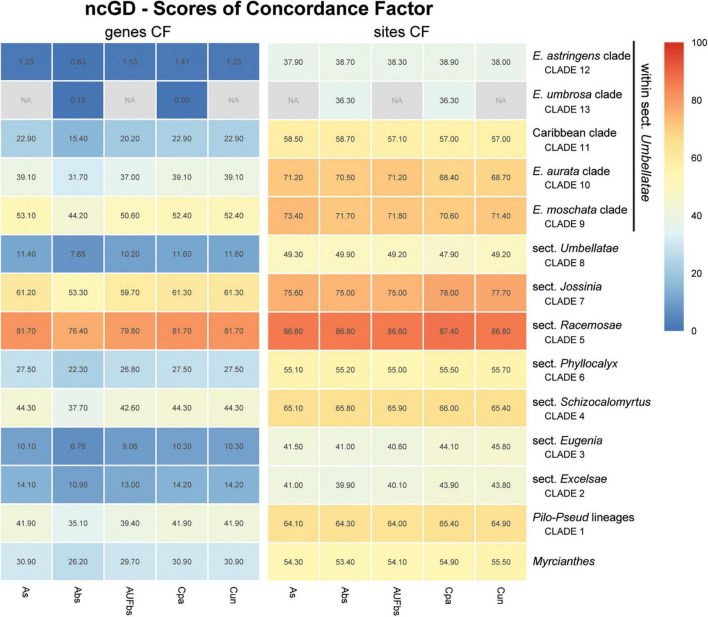
Heatmap of gene and site concordance factors (gCF and sCF) recovered in focus clades resulting from combination of nuclear coding and intronic non-coding loci in a genomic dataset (ncGD) of *Eugenia*. Each column is a method of phylogenetic reconstruction, and each row corresponds to a clade in Figures 2–4. Shading indicates percentage of concordance factor values recovered (Cpa, concatenated and partitioned ML reconstruction; Cun, concatenated and unpartitioned ML reconstruction; As, coalescent Astral tree with local posterior probability support; Abs, coalescent-based Astral tree with 100 bootstrap replicates; AUFbs, coalescent Astral tree with 1,000 ultrafast bootstrap replicates; NA, node not recovered).

**FIGURE 9:**
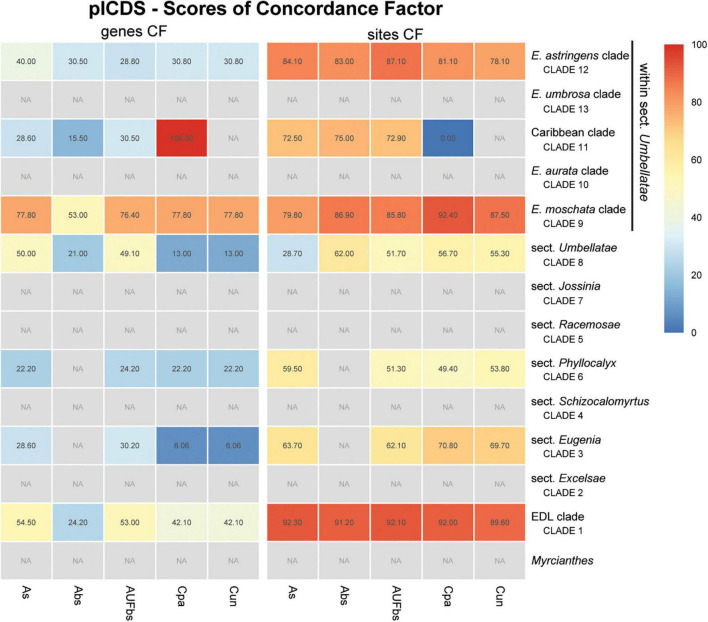
Heatmap of gene and site concordance factors (gCF and sCF) recovered in focus clades of the plastome coding loci (plCDS) in *Eugenia*. Each column is a method of phylogenetic reconstruction, and each row corresponds to a clade in Figures 2–4. Shading indicates percentage of concordance factor values recovered (Cpa, concatenated and partitioned ML reconstruction; Cun, concatenated and unpartitioned ML reconstruction; As, coalescent Astral tree with local posterior probability support; Abs, coalescent-based Astral tree with 100 bootstrap replicates; AUFbs, coalescent Astral tree with 1,000 ultrafast bootstrap replicates; NA, node not recovered).

Assessment of recurring nodes observed in the species trees focuses on 14 clades, one of which includes the outgroup *Myrcianthes*; seven correspond to the previously recognized sections of *Eugenia*, i.e., *Eugenia* sects. *Excelsae*, *Eugenia*, *Schizocalomyrtus*, *Racemosae*, *Jossinia*, *Phyllocalyx*, and *Umbellatae* (Figures 6–9); five clades are discussed within sect. *Umbellatae*; and one clade comprises two currently recognized sections (sects. *Pilothecium* and *Pseudeugenia*). The latter clade is sister to the remainder of *Eugenia* and is informally named as the *Pilo-Pseud* lineage (i.e., *Pilothecium*-*Pseudeugenia* lineage). Moderate scores of gene concordance factor (gCF) were recurrent in these lineages, in each of the three datasets (ncCDS, ncINT, and plCDS).

The ncCDS dataset returns contrasting gCF scores (Figure 6) and a general pattern of higher sCF. High discordance among genes is expressed by low values (cold colors) of gCF recovered for all the clades considered. *Eugenia* sects. *Racemosae* and *Jossinia* are the most remarkable exceptions, with moderate gCF (39.5–76.1%) and higher sCF (71.9–85.3%). Deeper nodes within *Umbellatae*, informally referred to here as the “*Eugenia aurata* clade” (clade 10), Caribbean clade (clade 11), “*E. astringens* clade” (clade 12), and “*E. umbrosa* clade” (clade 13), have low gCF and sCF scores (<27% and 35.9–65.9%, respectively). Regarding the method used for species tree reconstruction seen along the bottom of the heatmap, scores are similar. The only exceptions with slightly lower performance scores are the gCF of the Astral tree using bootstrap replicates (Abs), and in the sCF recovered with Astral with UFbs replicates (AUFbs). The ncINT dataset displays similar gCF and sCF scores for the “*Pilo-Pseud* lineages” (47.1–54.6% and 65.3–65.7%), *Eugenia* sect. *Schizocalomyrtus* (48–55.8 and 63.8–66.8%), sect. *Racemosae* (88.9–89.8 and 87–87.5%), sect. *Jossinia* (67.1–75.3 and 76–77.2%), “*Eugenia moschata* clade” (clade 9) (61.5–71.6 and 74.3–75.2%), and “*Eugenia aurata* clade” (clade 10) (43.6–54.5 and 73.8–74.5%) (Figure 7). Discordance among genes is mainly concentrated in *Eugenia* sects. *Eugenia*, *Excelsae*, and *Umbellatae*, especially in the deepest clades within the latter. Method of tree reconstruction has a minor influence on CF scores except in the concatenated trees (Cpa and Cun) that do not recover *Eugenia* sect. *Excelsae*. The ncGD dataset shows similar patterns of CF scores to those recovered from the ncCDS and ncINT datasets, but with intermediate scores as a result of the combination of both nuclear datasets (Figure 8). The off-target plCDS dataset recovered topologies lacking several clades (see NA in Figure 9) when contrasted with the nuclear datasets, recovering only *Eugenia* sects. *Eugenia*, *Phyllocalyx, Umbellatae*, and the “*Pilo-Pseud* lineages.” Clades within *Eugenia* sect. *Umbellatae*, such as the “*E. moschata* clade” (clade 9) (gCF 53–77.8% and sCF 79.8–92.4%), “Caribbean clade” (clade 11) (15.5–100 and 0–75%) and “*E. astringens* clade” (clade 12) (28.8–40 and 78.1–87.1%) mostly displayed moderate gCF scores. The “Caribbean clade” of the concatenated partitioned tree scored a maximum gCF (100%) and none following sCF (0%). Concatenated matrices appear more sensitive to reconstruction methods that performed less well.

## Discussion

The high quality of the data is the result of successful capture and sequencing of exon and intron contigs from the nuclear genome, providing phylogenetic reconstruction at the infrageneric level of *Eugenia* for the first time. This contrasts with previous phylogenetic reconstructions based on relatively few molecular markers, usually heavily reliant on mostly congruent plastid regions (Mazine et al., 2014, 2018; Bünger et al., 2016a; Giaretta et al., 2019b). However, some sections of *Eugenia* remain poorly resolved or received low support in these previous topologies, a consequence of insufficient polymorphism to overcome incongruent signal likely caused by incomplete lineage sorting (ILS) (Degnan and Rosenberg, 2009). The correlation of increased number of PIS with alignment length, particularly evident in the ncINT dataset, indicates that flanked introns concentrate valuable variability, now incorporated in the phylogenetic reconstructions. In addition, the plCDS data, by-product of the nuclear enrichment (e.g., Crowl et al., 2017; Villaverde et al., 2018) also provided a signal that is informative at several taxonomic levels, allowing recovery of additional and congruent phylogenetic relationships.

### Nuclear Versus Plastid Phylogenetic Reconstructions

Despite the data from the plastid genome only accounting for c. 3.5% of the total mapped reads and providing low levels of PIS relative to the nuclear dataset (Supplementary Figure 2), exons retrieved were sufficient to recover seven of the 14 focus clades in agreement with the nuclear dataset in most reconstructions (Figure 9). Intergenic regions of the off-target plastid genome have been shown to be of limited use to increase resolution compared to datasets based on exons (e.g., Villaverde et al., 2018; Murphy et al., 2020). In those studies, intron-flanking regions provided scattered sequences and ambiguous alignments providing low phylogenetic signal, findings that corroborate the decision not to include these data here. Discordance between the phylogenetic reconstruction of the plCDS dataset and published plastid-rich Sanger sequence-based phylogenies highlights the role of nuclear data in the resulting topologies (e.g., Mazine et al., 2014; Bünger et al., 2016a; Mazine et al., 2018; Giaretta et al., 2019b). In this study, conflicting signal between nuclear and plastid datasets is evident from both topology test (Table 3) and tree landscape (Figure 5) assessment. These diverging results can be partially explained by different genome evolutionary rates (Drouin et al., 2008; Smith, 2015), as nuclear genes with higher substitution rates accumulate more variation expressed as greater phylogenetic signal and resolution. This is particularly relevant for lineages such as *Eugenia*, in particular sect. *Umbellatae* that diverged relatively recently in the history of angiosperms, c. 27 mya (Vasconcelos et al., 2017).

### Inclusion of Intron and Intergenic Spacers

Concatenation of all loci is a common method for phylogenetic inference, but its use has been controversial because of statistical inconsistency (see Edwards, 2008; Roch and Steel, 2015). More recently, multispecies coalescent (MSC) methods (Mirarab et al., 2014; Mirarab and Warnow, 2015; Zhang et al., 2017) have been shown, using simulations, to yield more accurate species trees than concatenation methods under scenarios of ILS (Kubatko and Degnan, 2007; Mendes and Hahn, 2017). The “supergene” assumption of concatenation assumes a single species tree and ignores loci with different evolutionary histories resulting from gene duplication, horizontal gene transfer, lineage sorting, or even systematic error of overwhelming non-phylogenetic signal, leading to strong support for a potentially incorrect species tree (Jeffroy et al., 2006; Degnan and Rosenberg, 2009). Conflicts between the MSC approach and concatenation tree topologies are highlighted in the topology test that compares a fixed tree from the concatenated full partition scheme based on ncCDS with all the other 19 topologies. The more sensitive AU test shows that topologies based on ncCDS reconstructed using the MSC model were significantly different from the concatenated trees (Table 3). Concatenated and coalescent-based trees were different regardless of the nuclear dataset used. Although RF topology distribution clustered the trees according to the nuclear and plastid datasets (Figures 5A,B), the KC analysis better identified further segregation in the nuclear dataset that exclusively grouped Astral trees based on the ncINT and ncGD datasets (squares in Figures 5C,D). In particular, coalescence-based trees better accommodate the incongruence found in ncINT likely because of higher levels of PIS, while concatenated trees recover more conservative topologies, as they have a narrow distribution in the landscape. This suggests that the consistent phylogenetic relationships often identified in previous studies based on Sanger sequencing may be biased with the use of concatenated datasets (Mazine et al., 2014; Bünger et al., 2016a; Mazine et al., 2018; Giaretta et al., 2019b).

The Astral tree without replicates, based on both nuclear exons and introns in ncGD, exhibited intermediate dispersal within the distribution of all the Astral trees (Figure 5). This indicates that Astral successfully accommodates the weight of the high level of PIS in ncINT. Conversely, concatenated trees based on ncGD and ncINT clustered together, indicating limitations in this method to balance intron dominance in the topology. Concatenation method simulations (Song et al., 2012; Gatesy and Springer, 2013; Wu et al., 2013) suggest that these methods perform less well than coalescent based methods, or are more likely to produce inaccurate inferences with high support (e.g., Kubatko and Degnan, 2007; Degnan and Rosenberg, 2009; Heled and Drummond, 2010; Leaché and Rannala, 2011). The latter pattern appears to apply here, where phylogenies based on concatenated datasets show high support at nearly all nodes (Figures 2–4). However, congruence between concatenation and MSC tree topologies is remarkable, particularly at backbone nodes. This suggests a strong data signal in the history of *Eugenia* that promotes congruence. Differences in the topologies of concatenation and MSC approaches mostly result from the diverging phylogenetic position of *Eugenia* sects. *Jossinia*, *Phyllocalyx*, and *Racemosae*, and toward tips where most variation is expected. Additionally, short branches can be a source of poor performance in concatenated methods (Kubatko and Degnan, 2007; Salichos and Rokas, 2013). In *Eugenia*, short branches commonly subtend lineages sister to species-rich clades that comprise most of the extant diversity of the genus.

### Concatenated Versus Coalescent Analyses

Topologies produced from the ncCDS, ncINT, and ncGD datasets are similar except for minor differences in relationships of the focus clades (Figures 2–4). Moderate to weak support values are common at the backbone of Astral reconstructions, while strong support is more frequent in the concatenated trees. A possible explanation for these patterns may lie in the bootstrap statistic that depends on branch sampling variance, often low in large datasets (Felsenstein, 1985). As a result, high bootstrap values may result from resampling datasets that return the same topologies despite ILS or other processes of genealogical discordance (Minh et al., 2020), rather than from a genuinely strong phylogenetic signal. Alternatively, gene and site concordance factors (gCF and sCF) are expressed in percentages (Minh et al., 2020), and are used in conjunction with measures of statistical support to better recognize variation and sources of incongruence in the phylogenetic reconstructions. Values of statistical support can differ widely from the concordance factor. For instance, the deepest node of *Eugenia* in the ncCDS Astral tree is strongly supported (local pp:1), with a gCF of 37.4% and an sCF of 65.4%. This means that 114 loci of the 306 single locus trees recovered *Eugenia splendens* as sister to other *Eugenia* species (see Supplementary Figure 3.1), and that 65.4% of the informative sites for this branch support this arrangement. The relatively low gCF in comparison to sCF suggests that other stochastic processes besides conflicting signal are involved. This occurs frequently along the backbone of *Eugenia* where a general pattern of higher sCF versus gCF (Figure 8) indicates other sources of conflicting signal such as rapid radiation and recent divergence.

Concordance factors of ncINT (Figure 7) were shown to be substantially higher than those of the ncCDS (Figure 6), suggesting that combining these datasets in ncGD would improve phylogenetic reconstruction. Analysis of the heatmap based on ncGD shows that the degree of concordance increased in the focus clades in contrast to ncCDS (Figure 8). However, incorporation of ncINT, with high levels of PIS, in fact, had little impact on the resulting topologies, with support values decreasing in the coalescent based trees. The source of this genealogical disparity is likely related to highly variable intron regions where the phylogenetic signal may be better suited to resolve relationships at shallow phylogenetic levels (Weitemier et al., 2014; Johnson et al., 2016). Despite that, most focus clades are recovered in the phylogenies as result of a recurrent phylogenetic signal observed in loci of both the ncCDS and ncINT datasets.

*Eugenia* subg. *Eugenia* is shown to be monophyletic and strongly supported in the ncCDS and ncGD datasets (Figures 2, 4) and moderately supported in ncINT (Figure 3). However, *Eugenia* subg. *Pseudeugenia* was recovered only in the ML based on ncCDS but with low support (<0.75) (Figure 2). Short divergence times resulting in short branches (Pamilo and Nei, 1988; Salichos and Rokas, 2013) appear to be associated with low support at early diverging nodes, as also found in phylogenies recovered using Sanger sequence data (Mazine et al., 2014; Bünger et al., 2016a; Giaretta et al., 2019b). *Eugenia splendens* was repeatedly recovered as sister to all other *Eugenia*, while *E. dysenterica* was recovered as sister to *E.* subg. *Eugenia* in many reconstructions. This suggests that *Eugenia* subg. *Pseudeugenia* may not be monophyletic as currently circumscribed, consisting of two or more independent lineages. Additional evidence based on a large sample size will be necessary to confirm these patterns.

*Eugenia* sect. *Excelsae*, recently formally recognized by Mazine et al. (2018), was placed as sister to sect. *Schizocalomyrtus* (Mazine et al., 2014) in Sanger sequencing phylogenies, but with low support (Mazine et al., 2018; Giaretta et al., 2019b; Flickinger et al., 2020). The reconstructions presented here based on exons (ncCDS) recover sect. *Excelsae* as sister to sect. *Eugenia* (Figure 2) with moderate to weak support due to low levels of genealogical concordance (gCF = 0.5–1.88%). Two strongly supported clades emerge in *Eugenia* sect. *Eugenia*, as found in previous reconstructions (Mazine et al., 2014), refuting previous suggestions that this section might not be monophyletic (Mazine et al., 2018; Giaretta et al., 2019b).

*Eugenia* sect. *Schizocalomyrtus* is strongly supported and sister to a large clade that includes sects. *Jossinia*, *Racemosae*, *Phyllocalyx*, *Speciosae*, and *Umbellatae*. This placement is supported by ncCDS and coalescent approaches of ncINT and ncGD (Figures 2–4). Concatenated ncINT and ncGD instead recovered *Eugenia* sect. *Schizocalomyrtus* that is sister to sect. *Excelsae*, an arrangement also found in previous phylogenies (Mazine et al., 2014; Giaretta et al., 2019b; Flickinger et al., 2020). Alternatively, *Eugenia* sect. *Schizocalomyrtus* had been recovered as sister to sect. *Phyllocalyx* (Mazine et al., 2018). The lack of consensus among Sanger sequence topologies reflects low branching support around the placement of *Eugenia* sect. *Schizocalomyrtus*. The low support recovered in the concatenated ncGD analysis (Figure 4) suggests again that reconstruction method in combination with genealogical incongruence results in placement uncertainty. High-throughput sequence methods, such as those used, here appear to have captured a phylogenetic signal previously obscured by genealogical discordance and found a more reliable placement for *Eugenia* sect. *Schizocalomyrtus*.

*Eugenia* sect. *Jossinia*, one of the few lineages of Myrteae with an extra-Neotropical distribution, has been previously recovered as sister to sect. *Racemosae* (Mazine et al., 2018). Although the monophyly of sect. *Jossinia* is supported by moderate levels of genealogical concordance in ncCDS and ncINT (Figures 6, 7), the uncertainty remains regarding its phylogenetic position. Topologies based on the ncINT dataset and MSC analysis are inconclusive because of high levels of polytomies recovered, while trees from the concatenated data recovered *Eugenia* sect. *Jossinia* that is sister to sect. *Phyllocalyx*. A similar arrangement was returned by the ncCDS coalescent based trees, while in the concatenated trees *Eugenia* sect. *Jossinia* was recovered as sister to sect. *Speciosae* + *Umbellatae* (van der Merwe et al., 2005; Mazine et al., 2018; Giaretta et al., 2019b; Supplementary Figures 3.4,3.5). The uncertainty surrounding the placement of *Eugenia* sect. *Jossinia* appears related to a genuine discordant phylogenetic signal, suggested by similar scores of gene and site CF of ncINT (67.1–75.4 and 76–77.2%), also influenced by other processes as evidenced by the discrepancy of CF scores in ncCDS (39.5–50.5 and 71.9–73.1%).

*Eugenia* sect. *Phyllocalyx* was traditionally recognized by the convenient character of expanded and showy sepals at anthesis (Berg, 1857). However, a further non-sister lineage bearing showy sepals was recognized by molecular reconstructions, triggering segregation of the section into the currently accepted sects. *Phyllocalyx* and *Speciosae* (Bünger et al., 2016a,b). The phylogenomic data support this arrangement, with *Eugenia* sect. *Speciosae* strongly supported as sister to sect. *Umbellatae*. Uncertainty remains regarding the relationships of *Eugenia* sect. *Phyllocalyx*, but most reconstructions support it as sister to a clade containing sect. *Racemosae*.

*Eugenia* sect. *Racemosae* was circumscribed based on inflorescence with long racemes (Berg, 1856) and was supported as monophyletic by the phylogenetic reconstructions of Mazine et al. (2014, 2018) as well as the results discussed here. A clade formed by *Eugenia inversa* and *E. plicatocostata* emerges as sister to *Eugenia* sect. *Racemosae* (e.g., Mazine et al., 2014; Mazine et al., 2018; Giaretta et al., 2019b), but the lineages were maintained taxonomically distinct because of the non-racemose inflorescence of *Eugenia inversa*, expressed in fasciculate short axes (Mazine et al., 2018). The segregation of *Eugenia* sect. *Racemosae* and the clade formed by *Eugenia inversa* and *E. plicatocostata* is supported by low levels of concordance factors in both ncCDS and ncINT (gCF = 8.1–26.7 and sCF = 46.8–50.7%); it is possible that the latter clade may represent an until now unrecognized section.

### Systematic Relationships in *Eugenia* and *Eugenia* sect. *Umbellatae*

A lack of distinctive morphological patterns associated with clades and relationships with low statistical support have contributed to the “unmanageable” reputation of *Eugenia* sect. *Umbellatae* (Mazine et al., 2018). Those authors recovered seven clades within *Eugenia* sect. *Umbellatae*, also resolved here, along with five further clades of potential taxonomic significance (Figures 6–9). Target capture sequencing recovers a clade containing *Eugenia punicifolia* and *E. lagoensis* sister to the remaining species of *Eugenia* sect. *Umbellatae* in most coalescent-based reconstructions except for the concatenated trees. The results also suggest that *Eugenia melanogyna* is sister to the other species of *Eugenia* sect. *Umbellatae* as recovered in the ML trees (Mazine et al., 2018; Giaretta et al., 2019b). The “*E. moschata* clade” (clade 9) has been previously recovered (Mazine et al., 2018; Giaretta et al., 2019b) and, here, is statistically supported with mostly moderate gCF (ncCDS = 26.7–65.4% and ncINT = 61.5–75.2% and plCDS = 53–92.4%). Species of this clade are generally found in Amazon lowland forest and are morphologically united by partially fused sepals with membranous tissue beneath the seam joining the sepals (“membranisepalous” in Giaretta et al., 2019b).

Caribbean species were recently the focus of a comprehensive phylogenetic analysis that formally included previously segregated genera *Calyptrogenia* and *Hottea* within *Eugenia* (Flickinger et al., 2020). High-throughput sequencing strongly supports the “Caribbean clade,” recovered as sister to species distributed mostly in the Amazon and Guiana Shield lowland forest. From a biogeographical perspective, this relationship is more realistic than previous reconstructions that suggest an Atlantic forest origin of Caribbean lineages (Mazine et al., 2018; Giaretta et al., 2019b). Deeper in *Eugenia* sect. *Umbellatae*, there are two larger clades: the “*Eugenia astringens* clade” (12) and the “*Eugenia umbrosa* clade” (13) (Figure 8). Previous clades D and E of Mazine et al. (2018) correspond to the strongly supported clades 12/1 and 12/2, respectively (Figures 2–4). Here, clade 12/1 is sister to clade 12/2, in contrast with the Bayesian inference of Mazine et al. (2018) that recovered no close relationship between clades D and E. Species of clade 12/1 share similar aspects of inflorescence, i.e., fascicles with a variable axis length that often continue to grow after flowering and assume an auxotelic arrangement (Briggs and Johnson, 1979). Species of clade 12/2 share a tendency for inflorescences in short and congested fascicles. *Eugenia goiapabana* and *E. umbrosa* have been previously associated with clade D but, here, are placed in clade 13. Mazine et al. (2018) noted that *Eugenia goiapabana* and *E. umbrosa* are morphologically distinct from species that are placed here in clade 12/1 by their thick and large leaves and yellowish fruits. The MSC trees recover *Eugenia goiapabana* and *E. umbrosa* more closely related to *E. percincta*, also with large and thick leaves. The four lineages within clade 13 are well-supported, but relationships are uncertain because of phylogenetic discordance among them, hampering resolution in the concatenated and coalescent-based trees.

## Conclusion

The universal Angiosperm-353 target kit was successful in resolving relationships and disentangling genealogical incongruence in *Eugenia*, a fast-evolving and taxonomically complex lineage. Infrageneric groups previously recovered by Sanger sequencing were strongly supported by the species trees and gCF, but some questions remain where branches are shortest, especially in the early diverging lineages and deep within *Eugenia* sect. *Umbellatae*, where conflicting gene topologies prevent a strong phylogenetic signal. It is stressed here that high statistical support does not mean an overwhelming concordant signal and should be interpreted carefully especially in concatenated datasets. The general pattern of higher sCF than gCF in the backbone of *Eugenia* suggests stochastic processes. The analysis presented here does not support *Eugenia* subg. *Pseudeugenia* as currently circumscribed, or sect. *Pilothecium.* Further studies based on a wider taxonomic sample are necessary to determine if taxonomic change is required. Previous Sanger sequencing phylogenies all return short branches at the backbone of *Eugenia* sect. *Umbellatae* (Mazine et al., 2014; Giaretta et al., 2019b). In the context of splitting *Eugenia* sect. *Umbellatae* into workable clades, future studies should be focused on the source of the short branches responsible for severely misleading concatenated ML reconstructions (Kubatko and Degnan, 2007; Bryant and Hahn, 2020). Distance-based methods that generate phylogenetic networks could show where phylogenetic incongruence from ILS or admixture is present (Low et al. in prep.). Alternatively, statistical tests using internal branch lengths in tripled gene trees following MSC to distinguish between ILS and introgression (Edelman et al., 2019) are promising. Development of a specific bait kit for tribe Myrteae or *Eugenia* may also be useful to enhance resolution. Furthermore, we recommend additional targeted sequencing approaches with more species and individuals to enhance resolution. Targeted sequencing provides massive quantities of data that improve resolution, but uncertainty remains, likely because severe ILS at the deepest nodes of sect. *Umbellatae*, and pursuit of workable groups within sect. *Umbellatae* goes beyond increasing available data. Reconstruction methods must be carefully considered, as they influence topology significantly. The plastid genome is consistently better represented in Sanger sequencing reconstructions in *Eugenia*, and there is a significant difference among phylogenetic reconstructions based on the nuclear and plastid genomes. The analyses presented here rely heavily on the nuclear dataset that, on the whole, recovered topologies congruent with the “Sanger plastid trees” of the past, indicating that the phylogenetic signal in *Eugenia* is strongly expressed by both genomes. The off-target plastid dataset recovered here was inferior in terms of PIS in comparison to the targeted sequences. Despite this, some plastid-reconstructed clades were congruent with the exon topologies. This reinforces the importance of embracing as much data as possible and conducting exploratory analyses. Combining tree landscape with concordance factor scores, as that used here, is a robust and comprehensive approach that incorporates several reconstruction hypotheses. We suggest that this approach will inspire and enable more studies to perform targeted sequencing to investigate the evolutionary history of Myrtaceae.

## Data Availability Statement

The datasets generated for this study can be found at https://www.ncbi.nlm.nih.gov/bioproject/PRJNA797102.

## Author Contributions

AG, EL, and OM conceived the study. AG, BM, and OM performed lab work and phylogenomic analyses. AG and EL wrote the manuscript. All authors have edited and contributed to the writing and reviewed the manuscript.

## Conflict of Interest

The authors declare that the research was conducted in the absence of any commercial or financial relationships that could be construed as a potential conflict of interest.

## Publisher’s Note

All claims expressed in this article are solely those of the authors and do not necessarily represent those of their affiliated organizations, or those of the publisher, the editors and the reviewers. Any product that may be evaluated in this article, or claim that may be made by its manufacturer, is not guaranteed or endorsed by the publisher.
