# Supplementary material for: Phylogenetic Relationships Within the Hyper-Diverse Genus Eugenia (Myrtaceae: Myrteae) Based on Target Enrichment Sequencing
Source: Front Plant Sci. 2022 Feb 4;12:759460. doi: 10.3389/fpls.2021.759460 (PMC8855041; doi:10.3389/fpls.2021.759460)
Supplement: Supplementary file 1 [file Data_Sheet_1.zip › Supplementary Figure 2.DOCX]

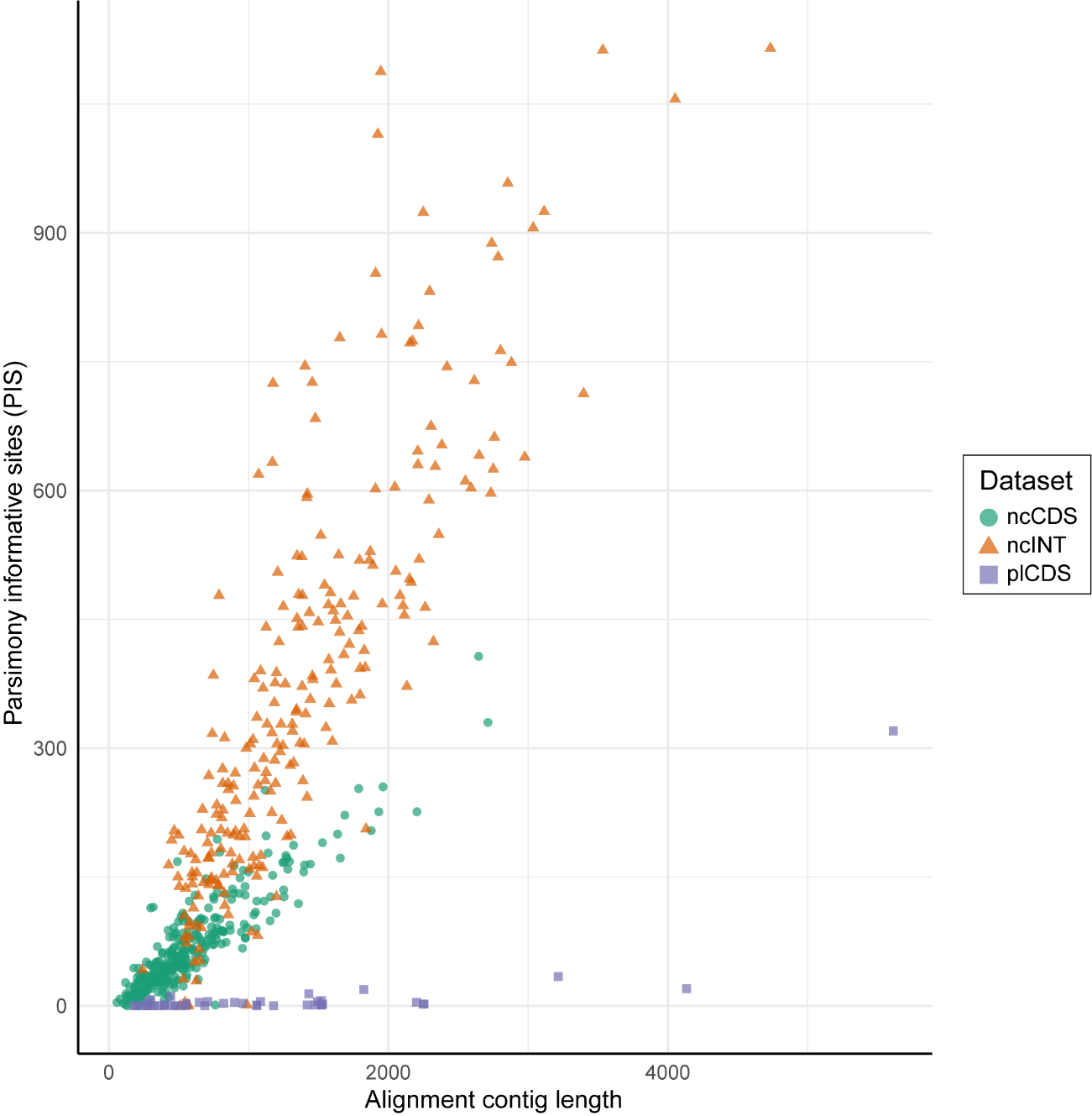


**Supplementary Figure 2.** Scatter plot of aligned contig length vs. number of parsimony informative sites (PIS) for the data generated for the three datasets recovered, targeting nuclear coding loci with the Angiosperm-353 probes in *Eugenia*.
