# Supplementary material for: Phylogenetic Relationships Within the Hyper-Diverse Genus Eugenia (Myrtaceae: Myrteae) Based on Target Enrichment Sequencing
Source: Front Plant Sci. 2022 Feb 4;12:759460. doi: 10.3389/fpls.2021.759460 (PMC8855041; doi:10.3389/fpls.2021.759460)
Supplement: Supplementary file 1 [file Data_Sheet_1.zip › Supplementary Figure 3.DOCX]

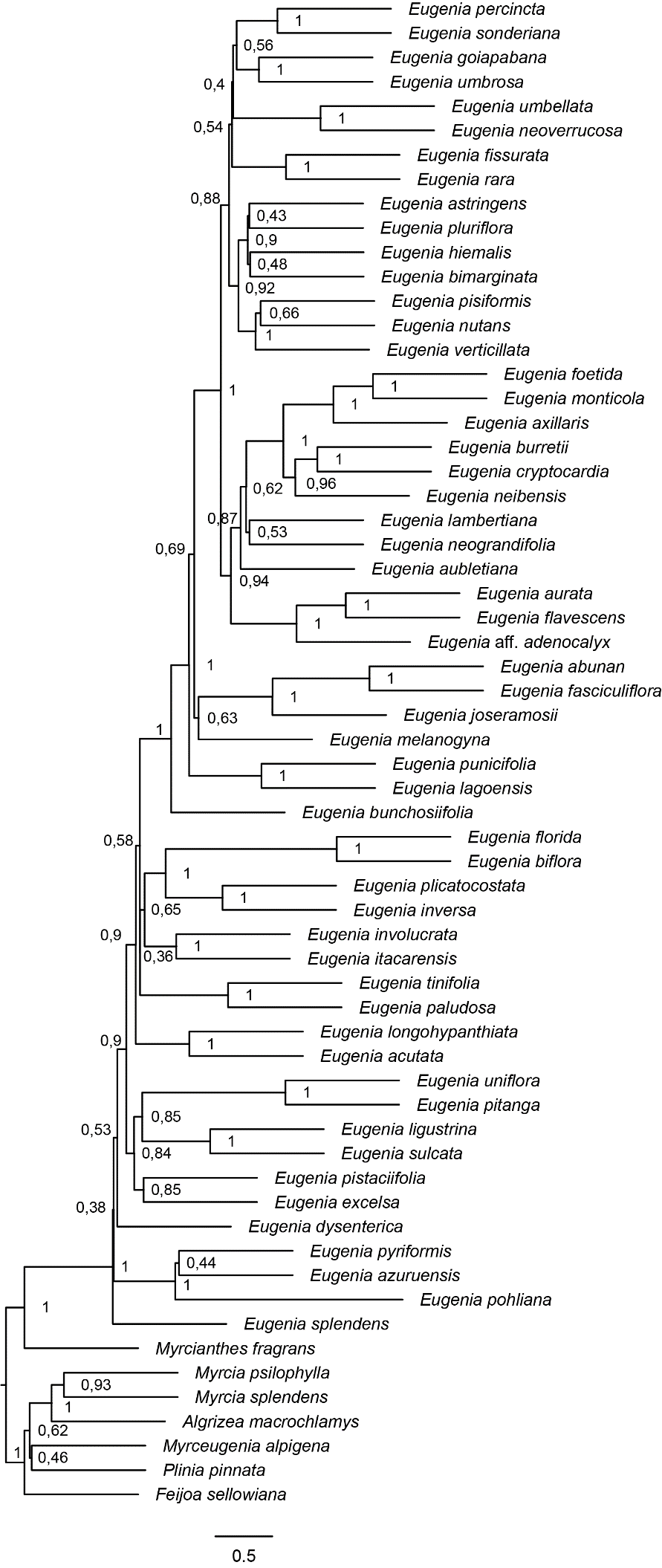


**Supplementary Figure 3.1.** Phylogenetic reconstruction of *Eugenia* based on 306 nuclear coding loci (ncCDS) targeted with the Angiosperm-353 probes. Multi-species coalescent approach tree using Astral with support values shown above branches (local pp/gCF/sCF). See ‘Material and Methods’ for a detailed description of the phylogenetic reconstruction. Dataset and analysis: ncCDS_As (See Table 1).


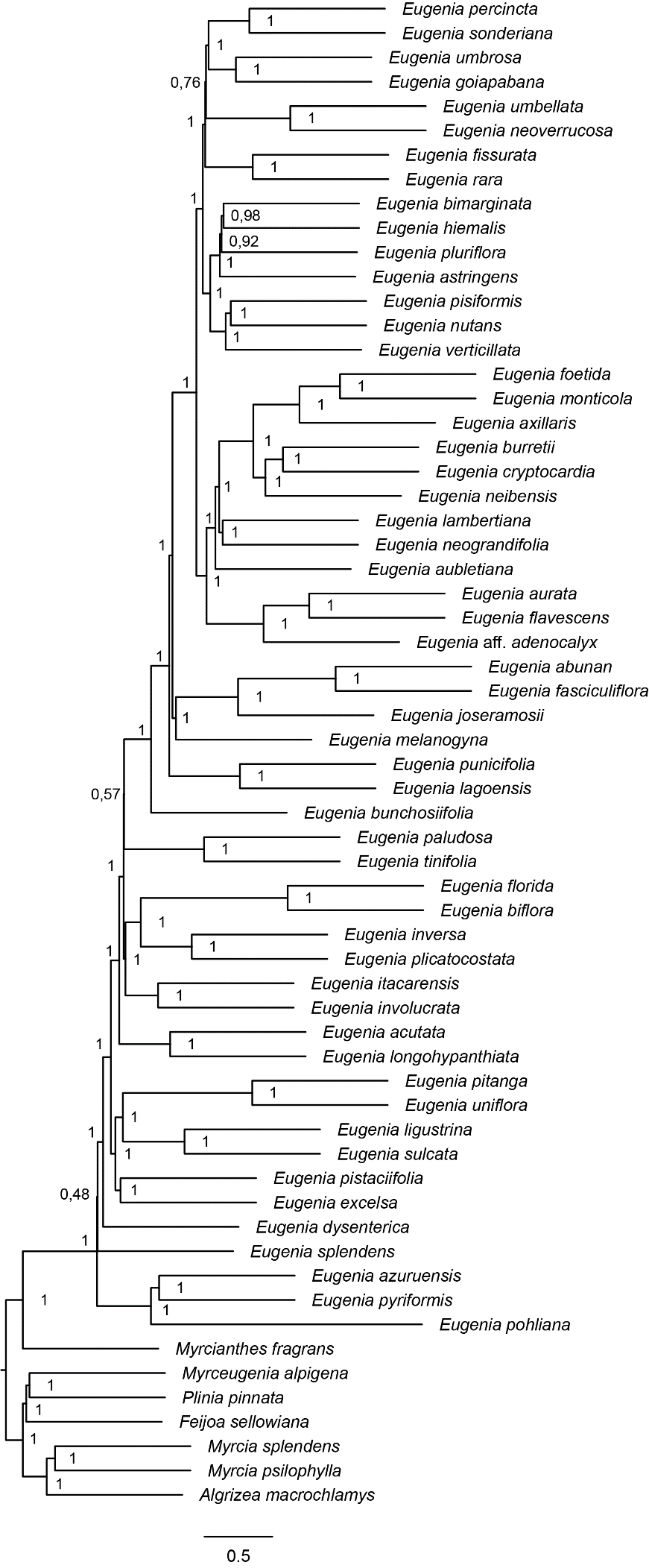


**Supplementary Figure 3.2.** Phylogenetic reconstruction of *Eugenia* based on 306 nuclear coding loci (ncCDS) targeted with the Angiosperm-353 probes. Multi-species coalescent approach tree using Astral with 100 bootstrap replicates for support values shown above branches (bootstrap/gCF/sCF). See ‘Material and Methods’ for a detailed description of the phylogenetic reconstruction. Dataset and analysis: ncCDS_Abs (See Table 1).


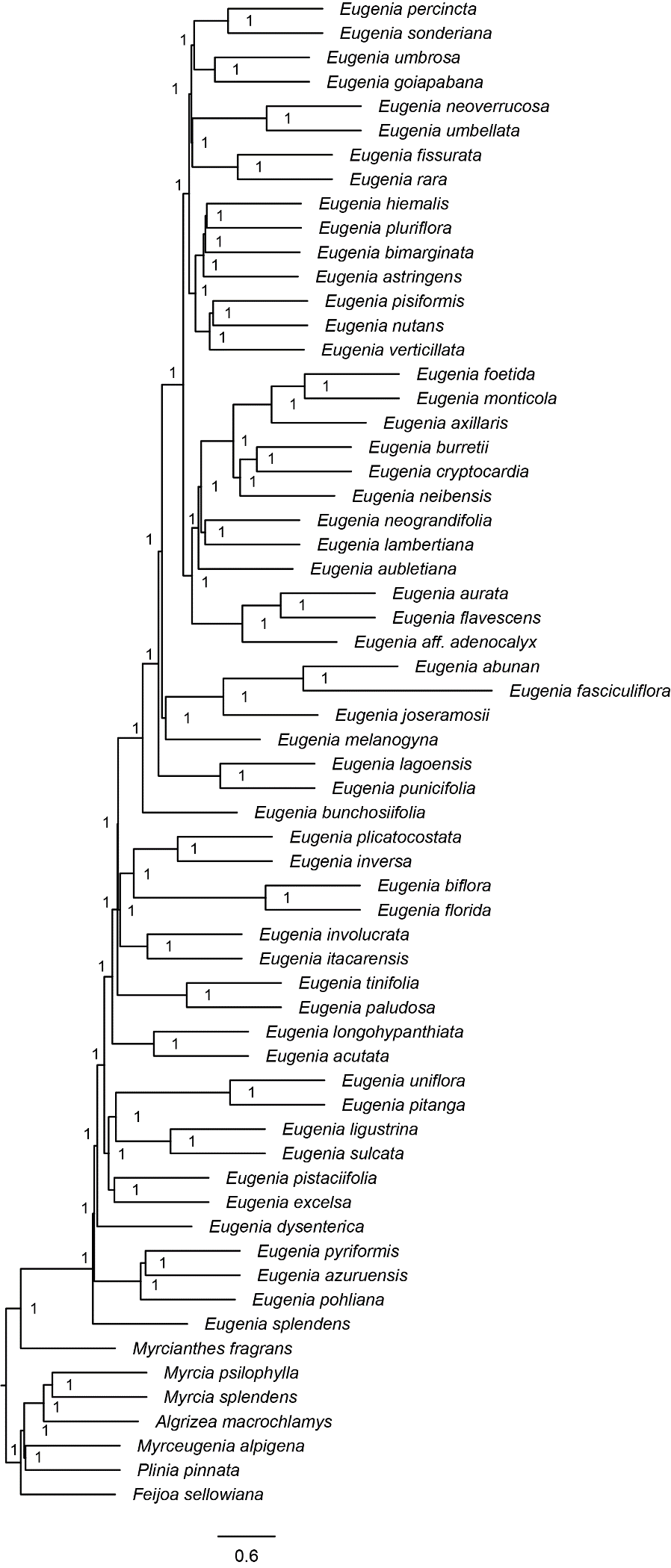


**Supplementary Figure 3.3.** Phylogenetic reconstruction of *Eugenia* based on 306 nuclear coding loci (ncCDS) targeted with the Angiosperm-353 probes. Multi-species coalescent approach tree using Astral with 1000 ultrafast bootstrap replicates for support values shown above branches (UFbs/gCF/sCF). See ‘Material and Methods’ for a detailed description of the phylogenetic reconstruction. Dataset and analysis: ncCDS_AUFbs (See Table 1).


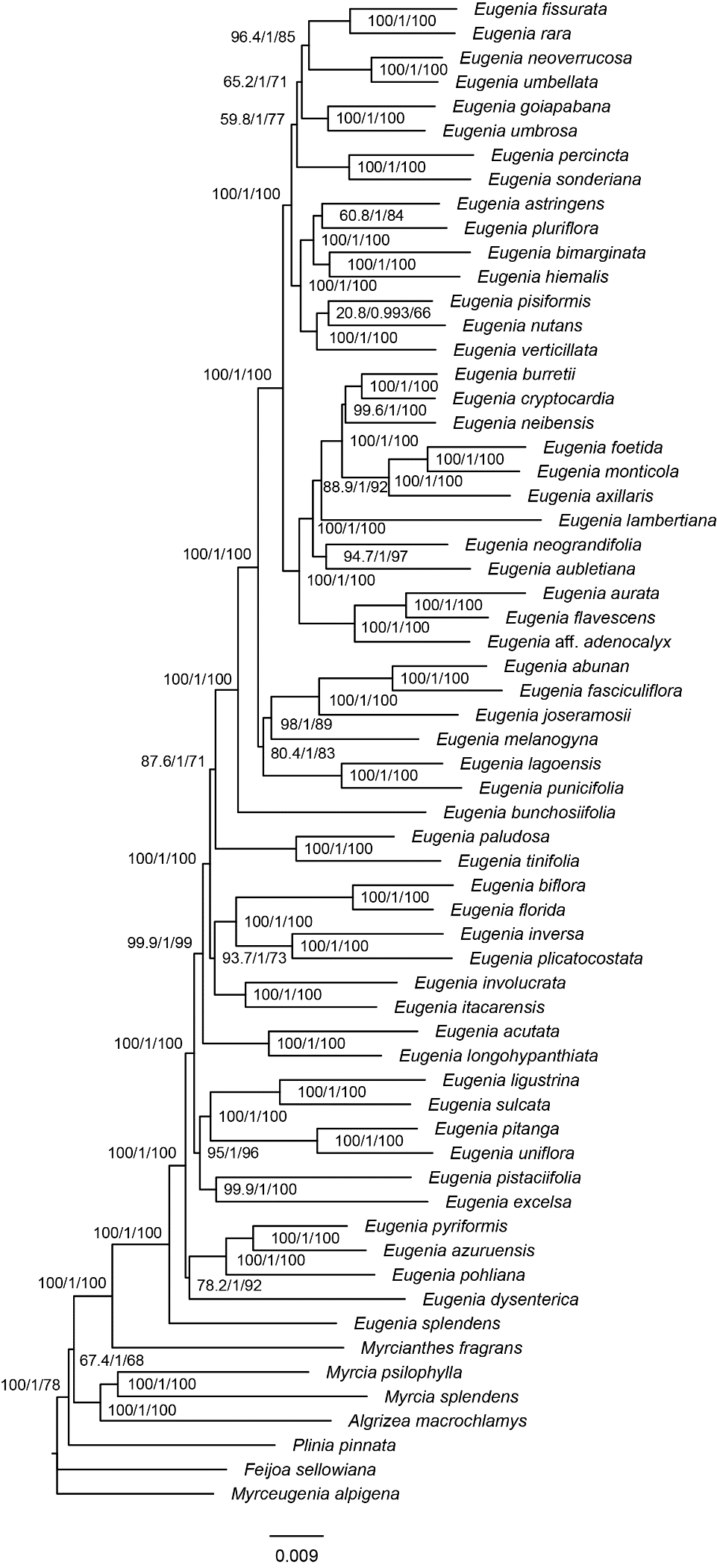


**Supplementary Figure 3.4.** Phylogenetic reconstruction of *Eugenia* based on 306 nuclear coding loci (ncCDS) targeted with the Angiosperm-353 probes. Maximum Likelihood concatenated partitioned tree with bootstrap support at the nodes and additional tests to branch support with values above branches (bootstrap/aBayes /SH-aLRT /gCF/sCF). See ‘Material and Methods’ for a detailed description of the phylogenetic reconstruction. Dataset and analysis: ncCDS_Cpa (See Table 1).


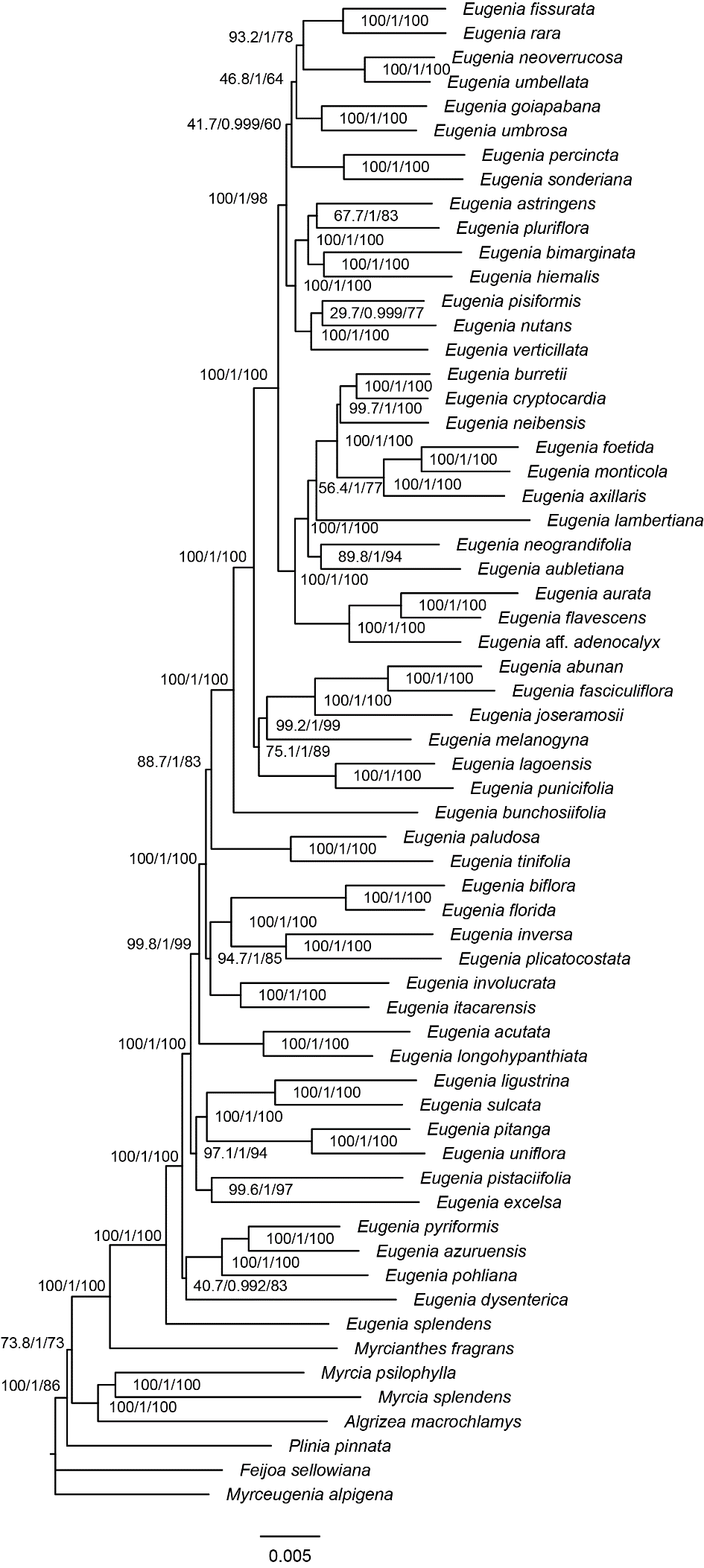


**Supplementary Figure 3.5.** Phylogenetic reconstruction of *Eugenia* based on 306 nuclear coding loci (ncCDS) targeted with the Angiosperm-353 probes. Maximum Likelihood concatenated unpartitioned tree with bootstrap support at the nodes and additional tests to branch support with values above branches (bootstrap/aBayes /SH-aLRT /gCF/sCF). See ‘Material and Methods’ for a detailed description of the phylogenetic reconstruction. Dataset and analysis: ncCDS_Cun (See Table 1).


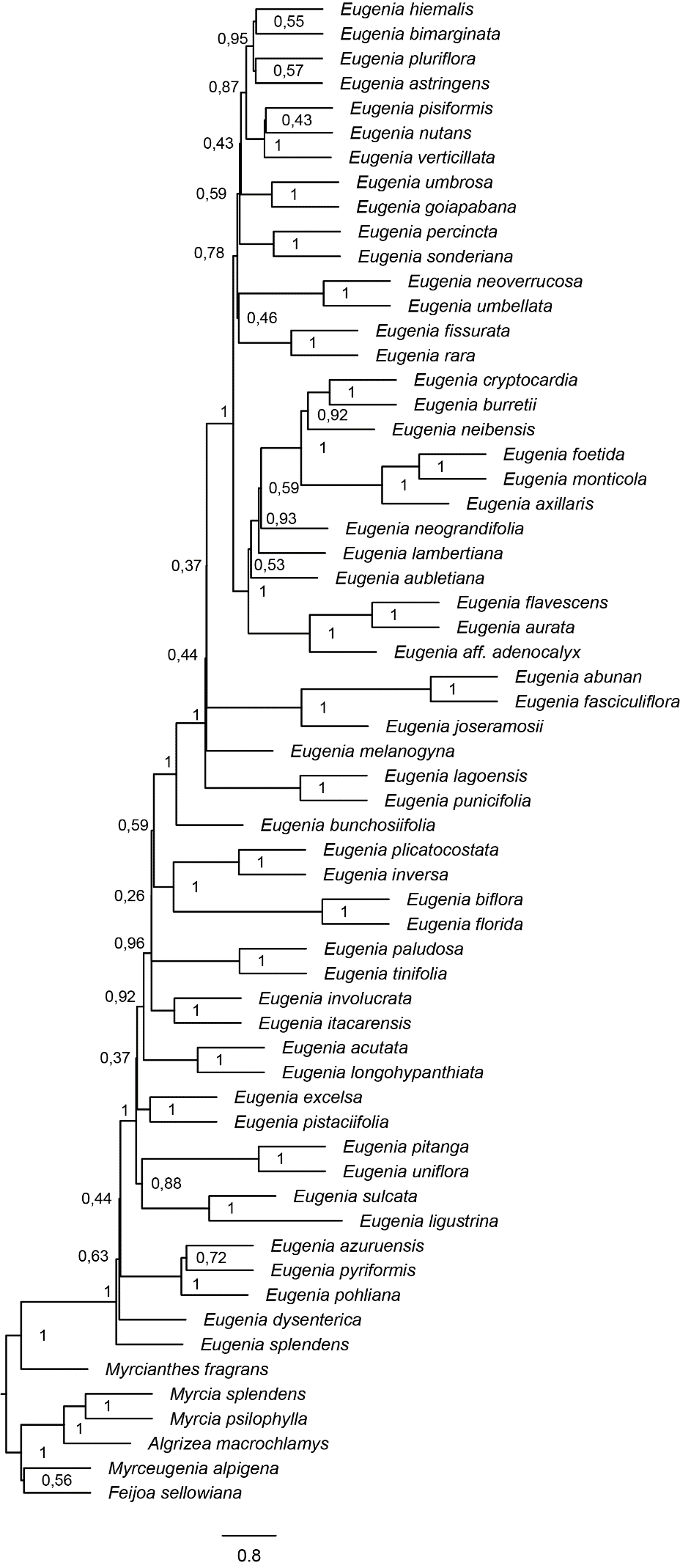


**Supplementary Figure 3.6.** Phylogenetic reconstruction of *Eugenia* based on 239 nuclear non-coding loci (ncINT) targeted with the Angiosperm-353 probes. Multi-species coalescent approach tree using Astral with support values shown above branches (local pp/gCF/sCF). See ‘Material and Methods’ for a detailed description of the phylogenetic reconstruction. Dataset and analysis: ncINT_As (See Table 1).


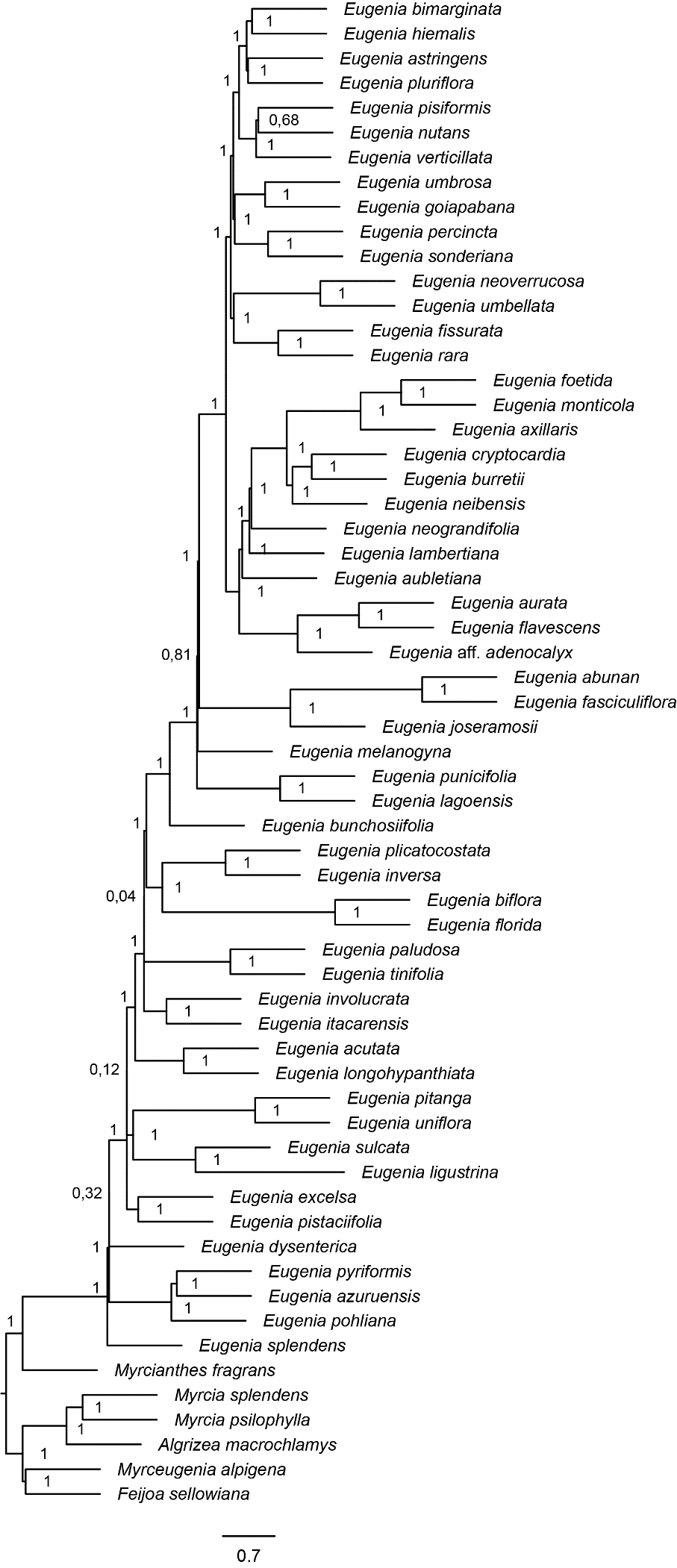


**Supplementary Figure 3.7.** Phylogenetic reconstruction of *Eugenia* based on 239 nuclear non-coding loci (ncINT) targeted with the Angiosperm-353 probes. Multi-species coalescent approach tree using Astral with 100 bootstrap replicates for support values shown above branches (bootstrap/gCF/sCF). See ‘Material and Methods’ for a detailed description of the phylogenetic reconstruction. Dataset and analysis: ncINT_Abs (See Table 1).


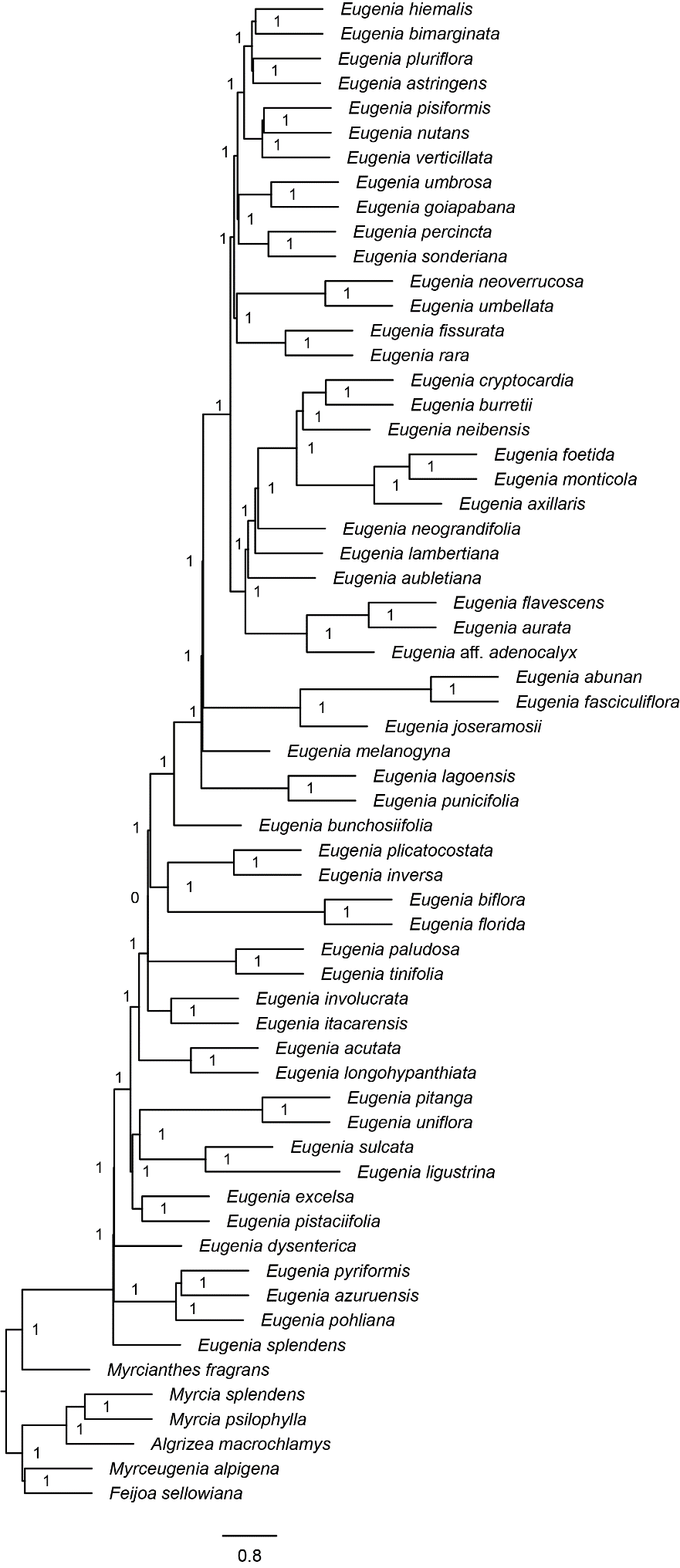


**Supplementary Figure 3.8.** Phylogenetic reconstruction of *Eugenia* based on 239 nuclear non-coding loci (ncINT) targeted with the Angiosperm-353 probes. Multi-species coalescent approach tree using Astral with 1000 ultrafast bootstrap replicates for support values shown above branches (UFbs/gCF/sCF). See ‘Material and Methods’ for a detailed description of the phylogenetic reconstruction. Dataset and analysis: ncINT_AUFbs (See Table 1).


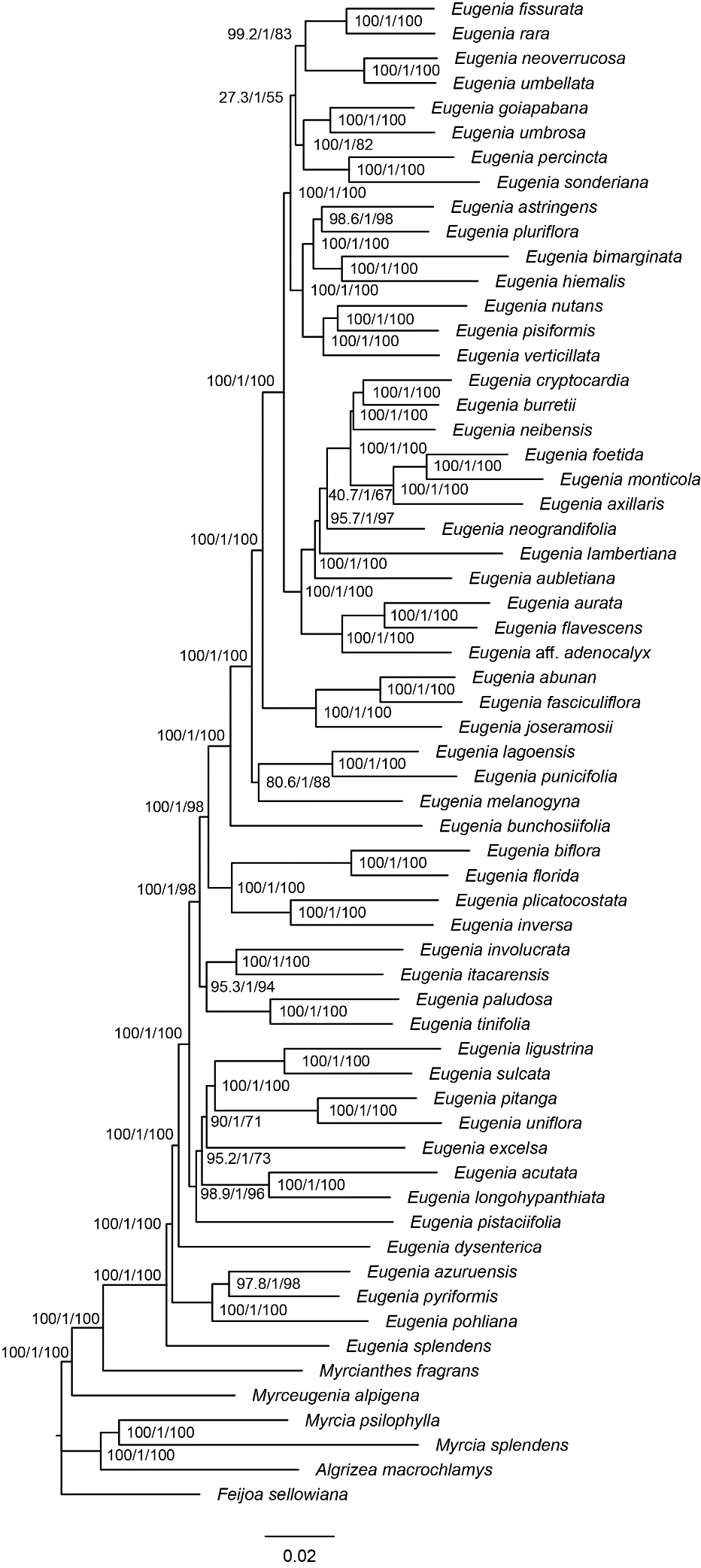


**Supplementary Figure 3.9.** Phylogenetic reconstruction of *Eugenia* based on 239 nuclear non-coding loci (ncINT) targeted with the Angiosperm-353 probes. Maximum Likelihood concatenated partitioned tree with bootstrap support at the nodes and additional tests to branch support with values above branches (bootstrap/aBayes /SH-aLRT /gCF/sCF). See ‘Material and Methods’ for a detailed description of the phylogenetic reconstruction. Dataset and analysis: ncINT_Cpa (See Table 1).


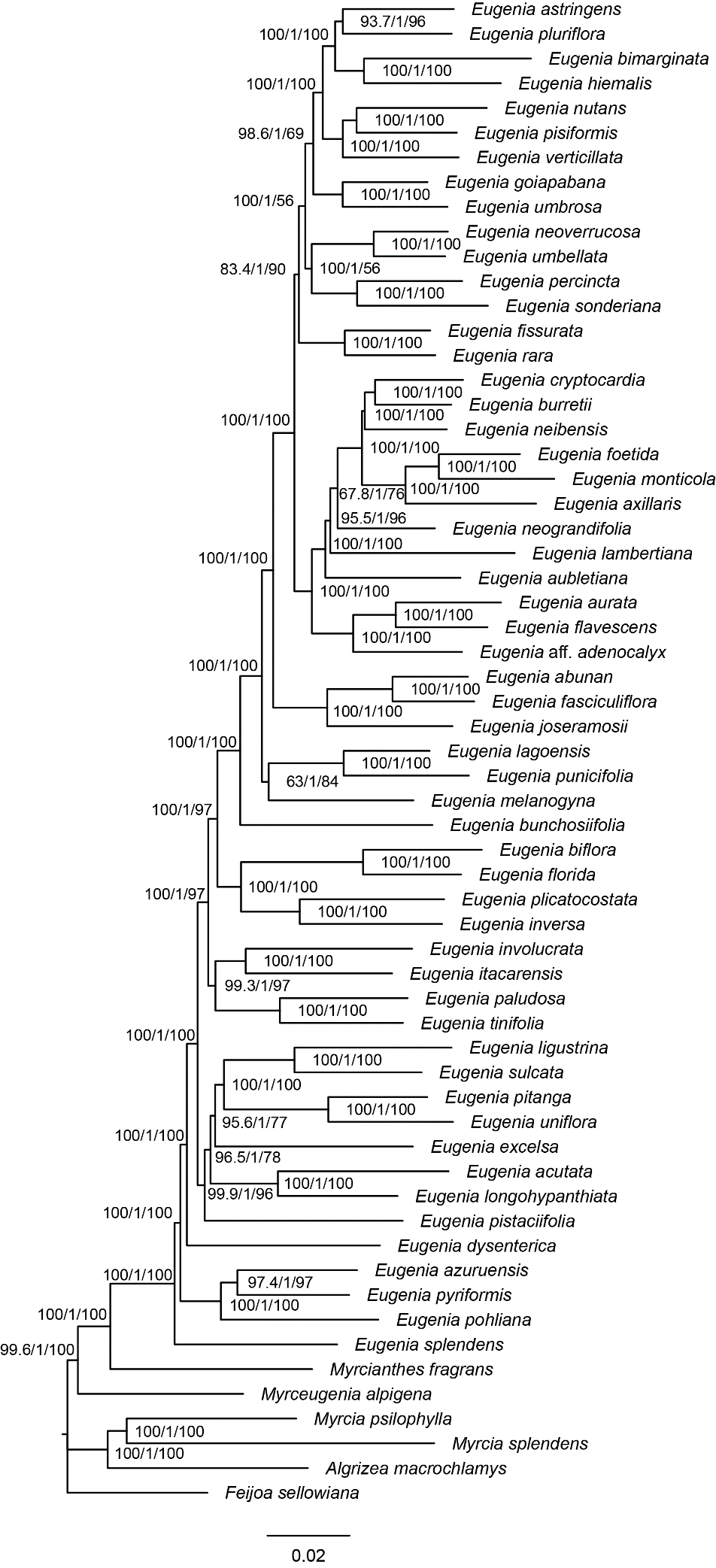


**Supplementary Figure 3.10.** Phylogenetic reconstruction of *Eugenia* based on 239 nuclear non-coding loci (ncINT) targeted with the Angiosperm-353 probes. Maximum Likelihood concatenated unpartitioned tree with bootstrap support at the nodes and additional tests to branch support with values above branches (bootstrap/aBayes /SH-aLRT /gCF/sCF). See ‘Material and Methods’ for a detailed description of the phylogenetic reconstruction. Dataset and analysis: ncINT_Cun (See Table 1).


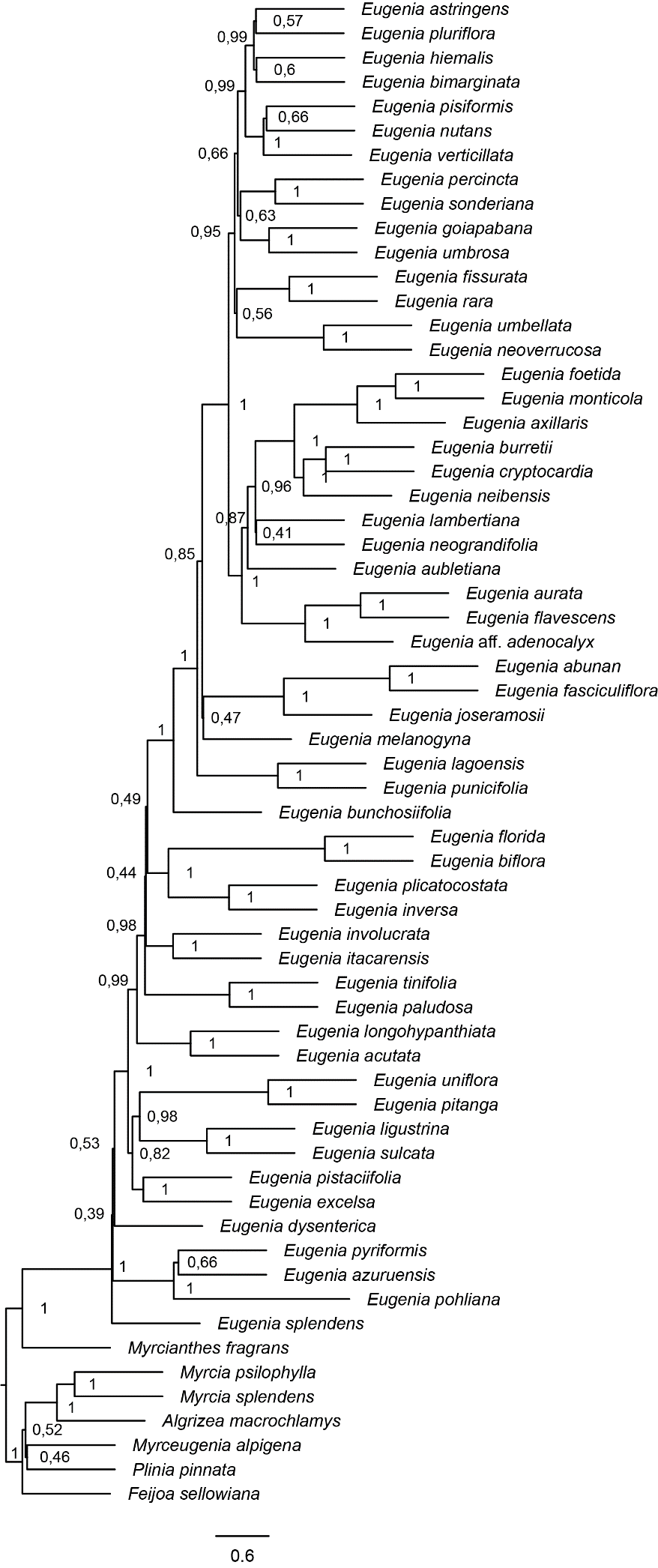


**Supplementary Figure 3.11.** Phylogenetic reconstructions of *Eugenia* based on the 306 nuclear coding loci with 239 additional intron regions targeted with the Angiosperm-353 probes combined in a genomic dataset (ncGD). Multi-species coalescent approach tree using Astral with support values shown above branches (local pp/gCF/sCF). See ‘Material and Methods’ for a detailed description of the phylogenetic reconstruction. Dataset and analysis: ncGD_As (See Table 1).


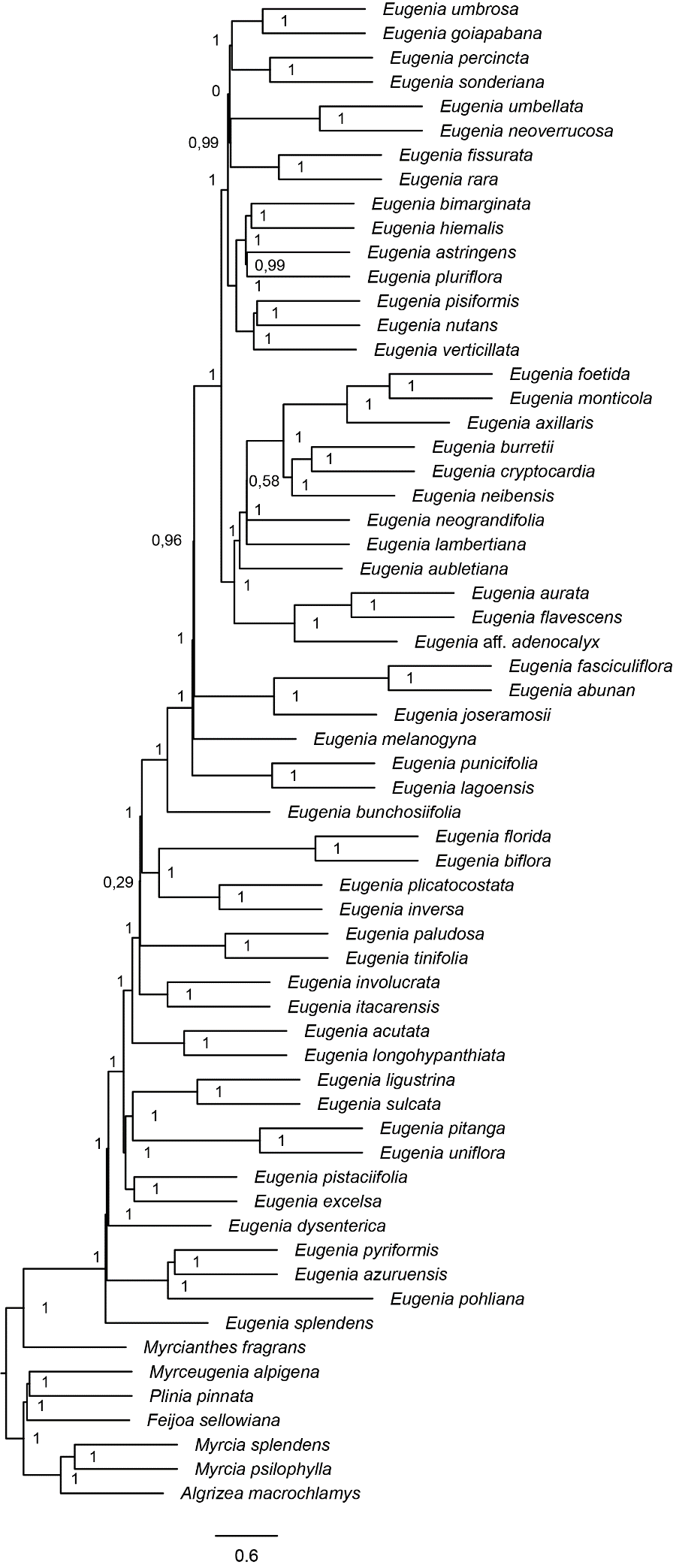


**Supplementary Figure 3.12.** Phylogenetic reconstructions of *Eugenia* based on the 306 nuclear coding loci with 239 additional intron regions targeted with the Angiosperm-353 probes combined in a genomic dataset (ncGD). Multi-species coalescent approach tree using Astral with 100 bootstrap replicates for support values shown above branches (bootstrap/gCF/sCF). See ‘Material and Methods’ for a detailed description of the phylogenetic reconstruction. Dataset and analysis: ncGD_Abs (See Table 1).


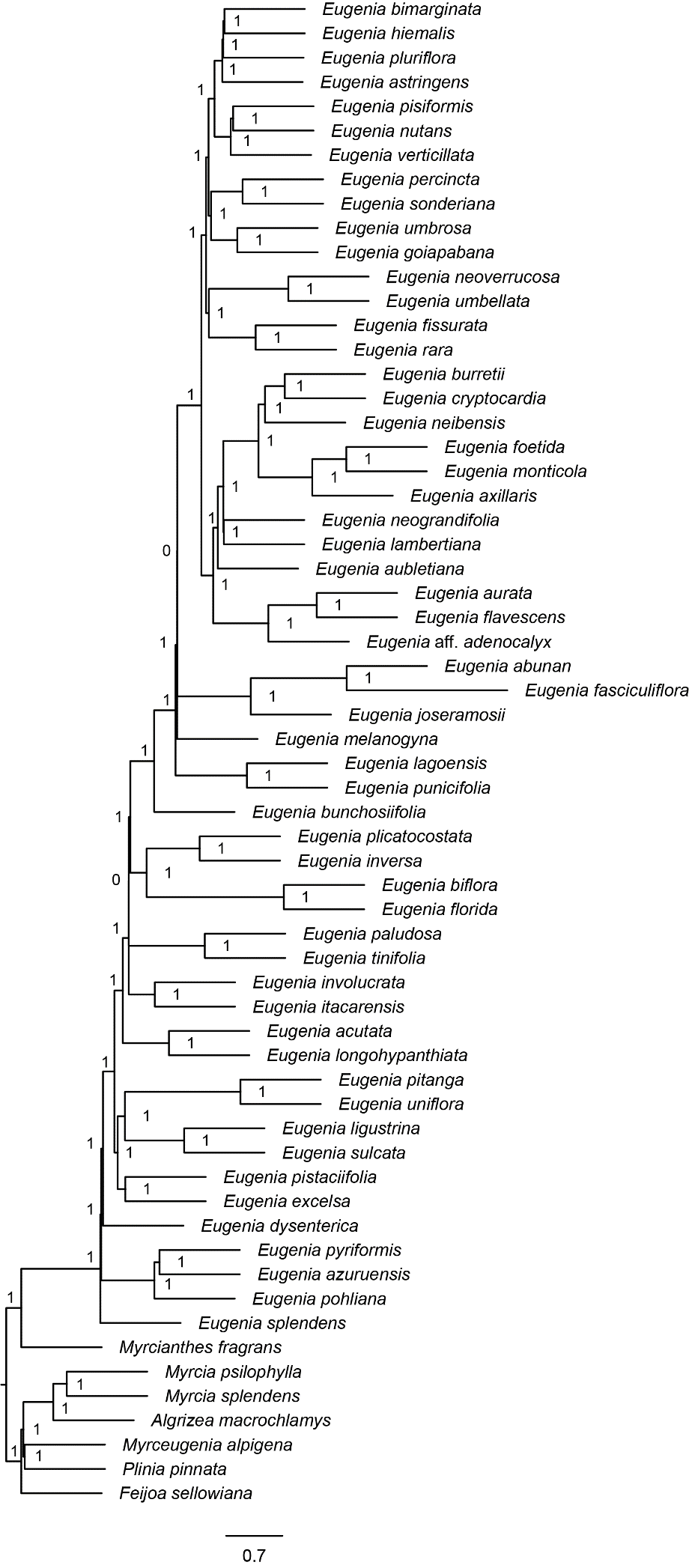


**Supplementary Figure 3.13.** Phylogenetic reconstructions of *Eugenia* based on the 306 nuclear coding loci with 239 additional intron regions targeted with the Angiosperm-353 probes combined in a genomic dataset (ncGD). Multi-species coalescent approach tree using Astral with 1000 ultrafast bootstrap replicates for support values shown above branches (UFbs/gCF/sCF). See ‘Material and Methods’ for a detailed description of the phylogenetic reconstruction. Dataset and analysis: ncGD_AUFbs (See Table 1).


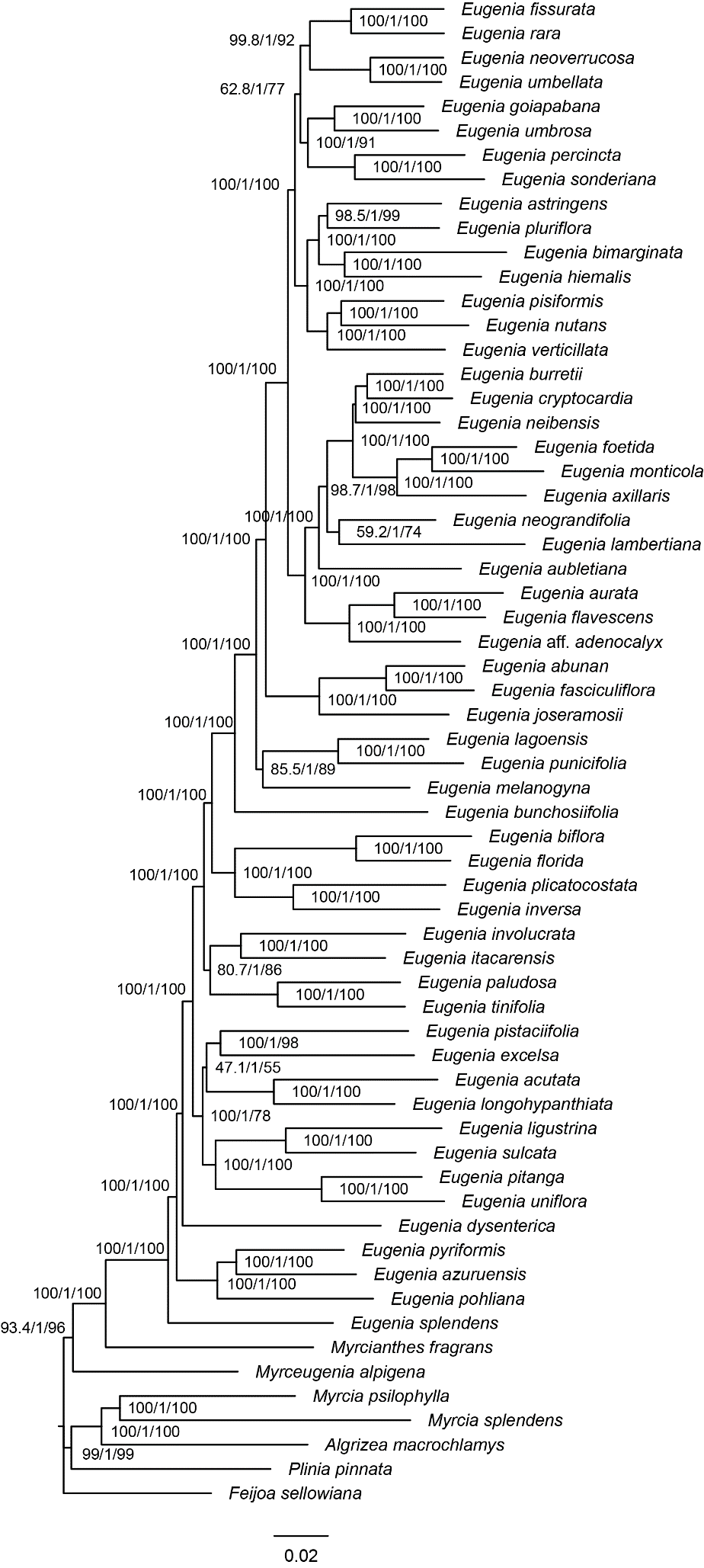


**Supplementary Figure 3.14.** Phylogenetic reconstructions of *Eugenia* based on the 306 nuclear coding loci with 239 additional intron regions targeted with the Angiosperm-353 probes combined in a genomic dataset (ncGD). Maximum Likelihood concatenated partitioned tree with bootstrap support at the nodes and additional tests to branch support with values above branches (bootstrap/aBayes /SH-aLRT /gCF/sCF). See ‘Material and Methods’ for a detailed description of the phylogenetic reconstruction. Dataset and analysis: ncGD_Cpa (See Table 1).


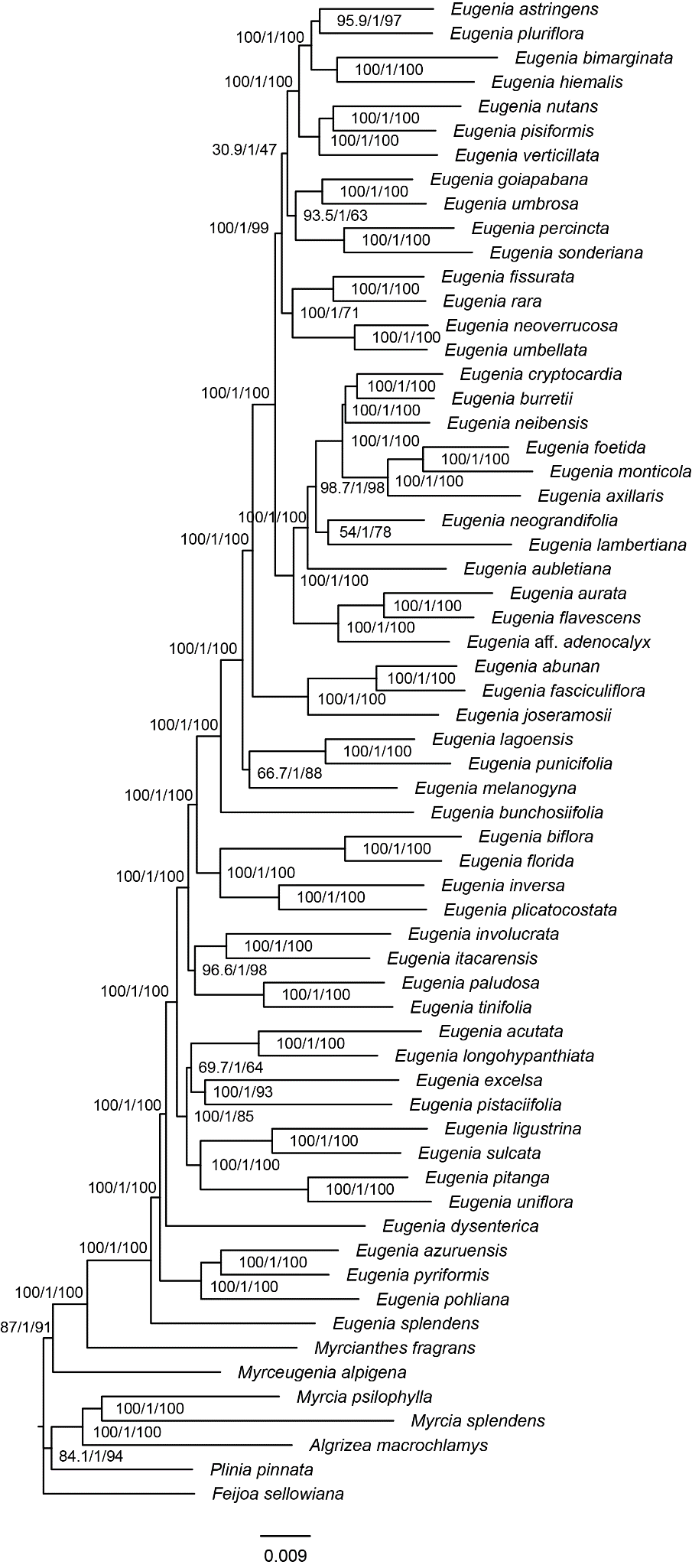


**Supplementary Figure 3.15.** Phylogenetic reconstructions of *Eugenia* based on the 306 nuclear coding loci with 239 additional intron regions targeted with the Angiosperm-353 probes combined in a genomic dataset (ncGD). Maximum Likelihood concatenated unpartitioned tree with bootstrap support at the nodes and additional tests to branch support with values above branches (bootstrap/aBayes /SH-aLRT /gCF/sCF). See ‘Material and Methods’ for a detailed description of the phylogenetic reconstruction. Dataset and analysis: ncGD_Cun (See Table 1).


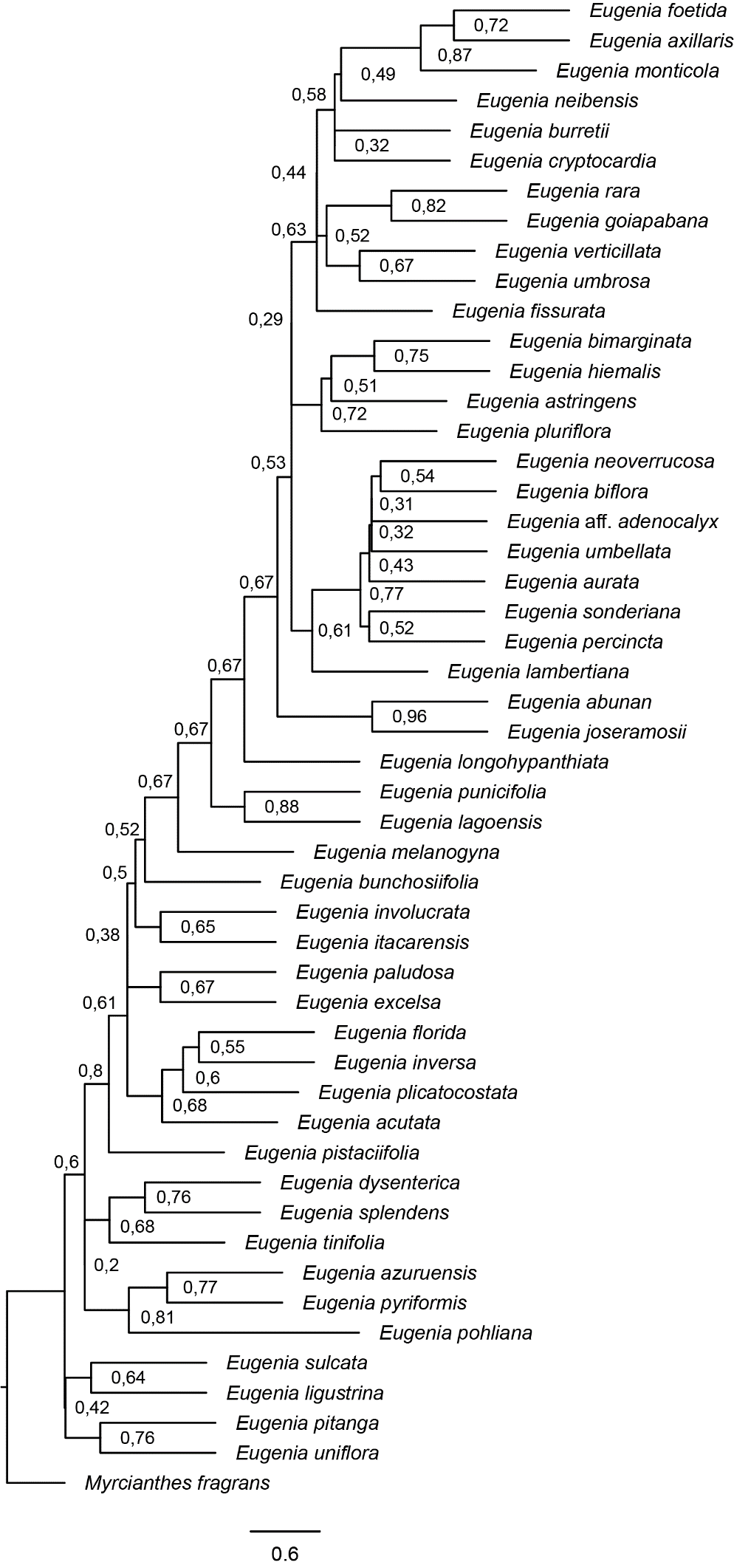


**Supplementary Figure 3.16.** Phylogenetic reconstruction of *Eugenia* based on the 44 plastome coding loci (plCDS) targeted with the Angiosperm-353 probes. Multi-species coalescent approach tree using Astral with support values shown above branches (local pp/gCF/sCF). See ‘Material and Methods’ for a detailed description of the phylogenetic reconstruction. Dataset and analysis: plCDS_As (See Table 1).


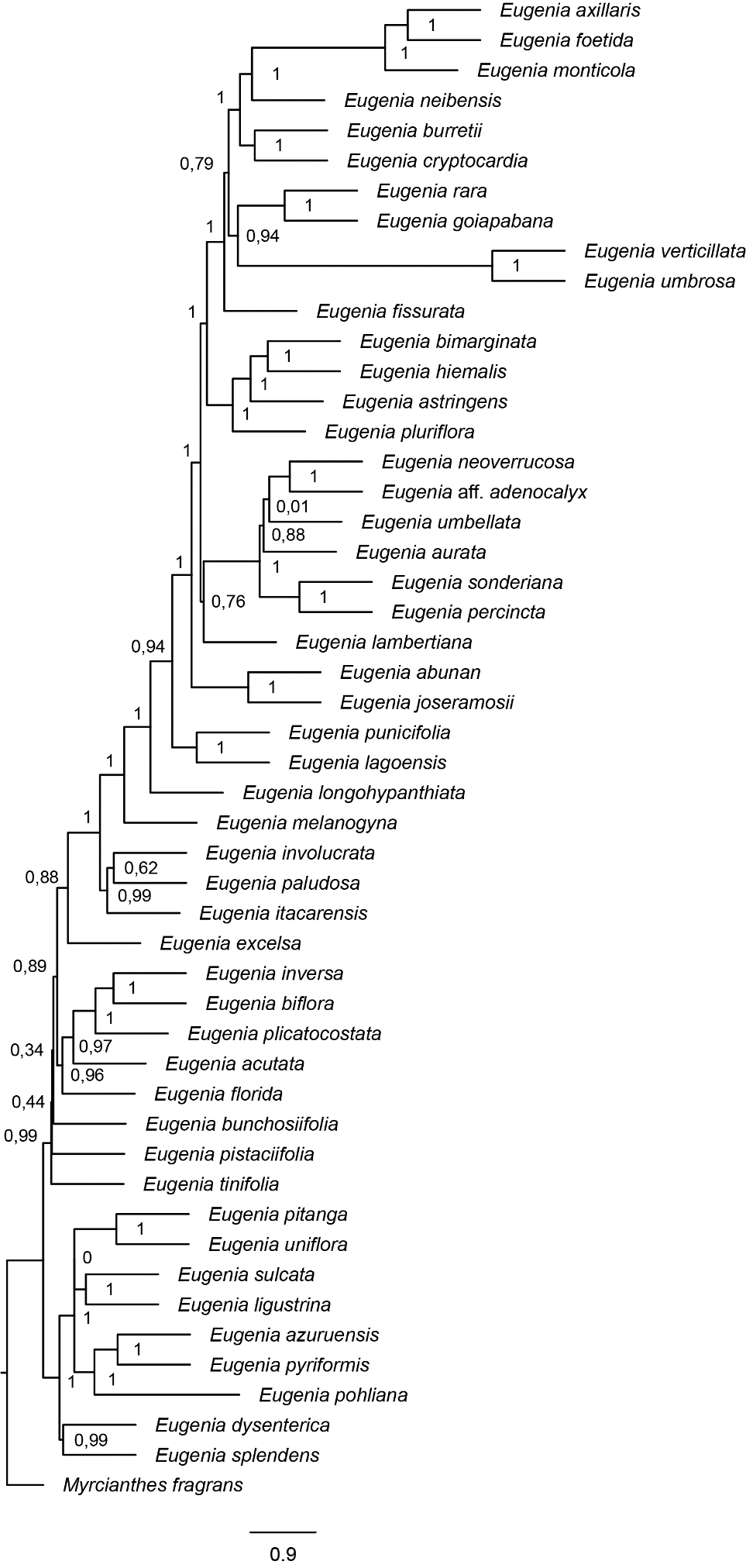


**Supplementary Figure 3.17.** Phylogenetic reconstruction of *Eugenia* based on the 44 plastome coding loci (plCDS) targeted with the Angiosperm-353 probes. Multi-species coalescent approach tree using Astral with 100 bootstrap replicates for support values shown above branches (bootstrap/gCF/sCF). See ‘Material and Methods’ for a detailed description of the phylogenetic reconstruction. Dataset and analysis: plCDS_Abs (See Table 1).


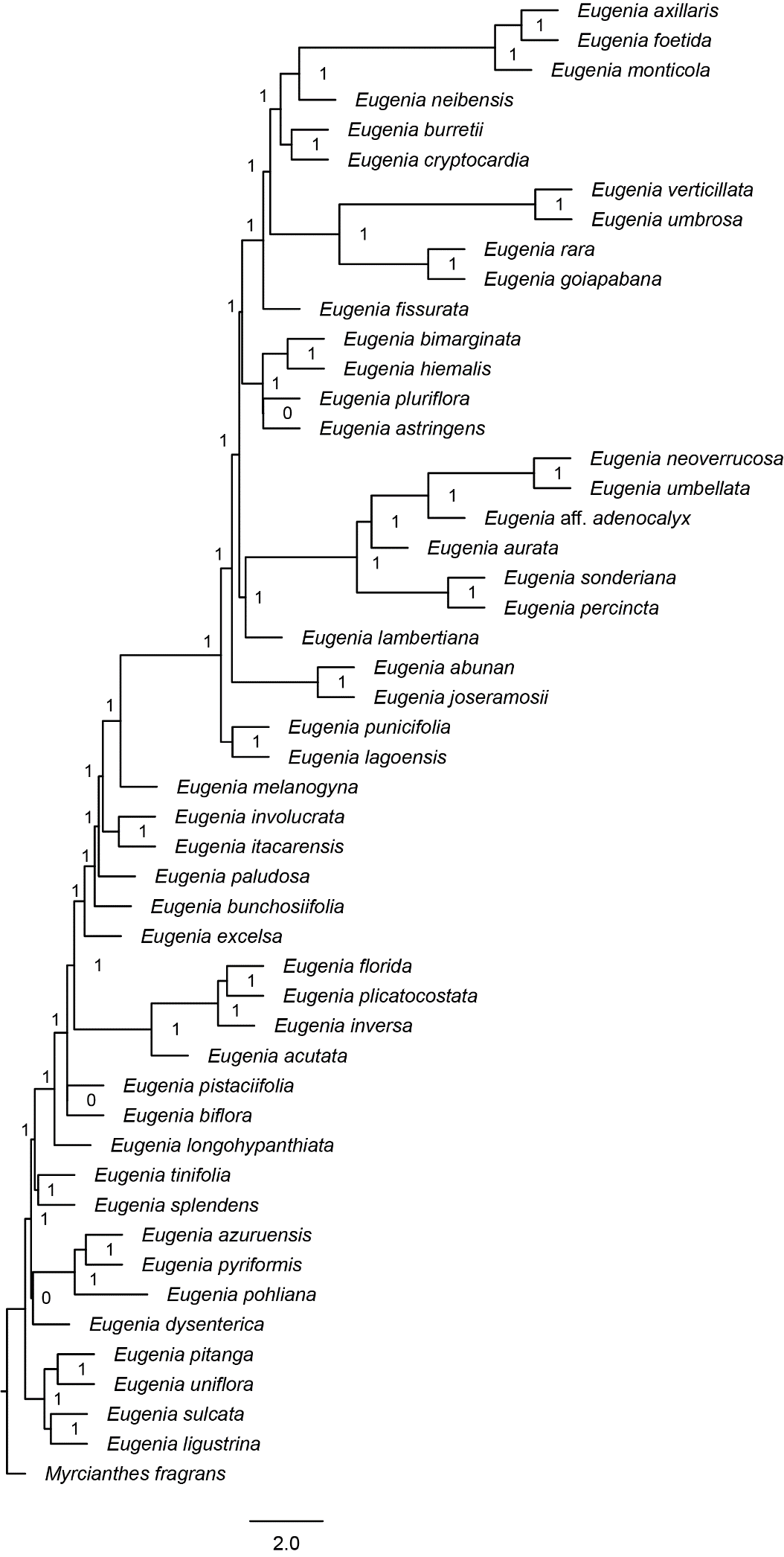


**Supplementary Figure 3.18.** Phylogenetic reconstruction of *Eugenia* based on the 44 plastome coding loci (plCDS) targeted with the Angiosperm-353 probes. Multi-species coalescent approach tree using Astral with 1000 ultrafast bootstrap replicates for support values shown above branches (UFbs/gCF/sCF). See ‘Material and Methods’ for a detailed description of the phylogenetic reconstruction. Dataset and analysis: plCDS_AUFbs (See Table 1).


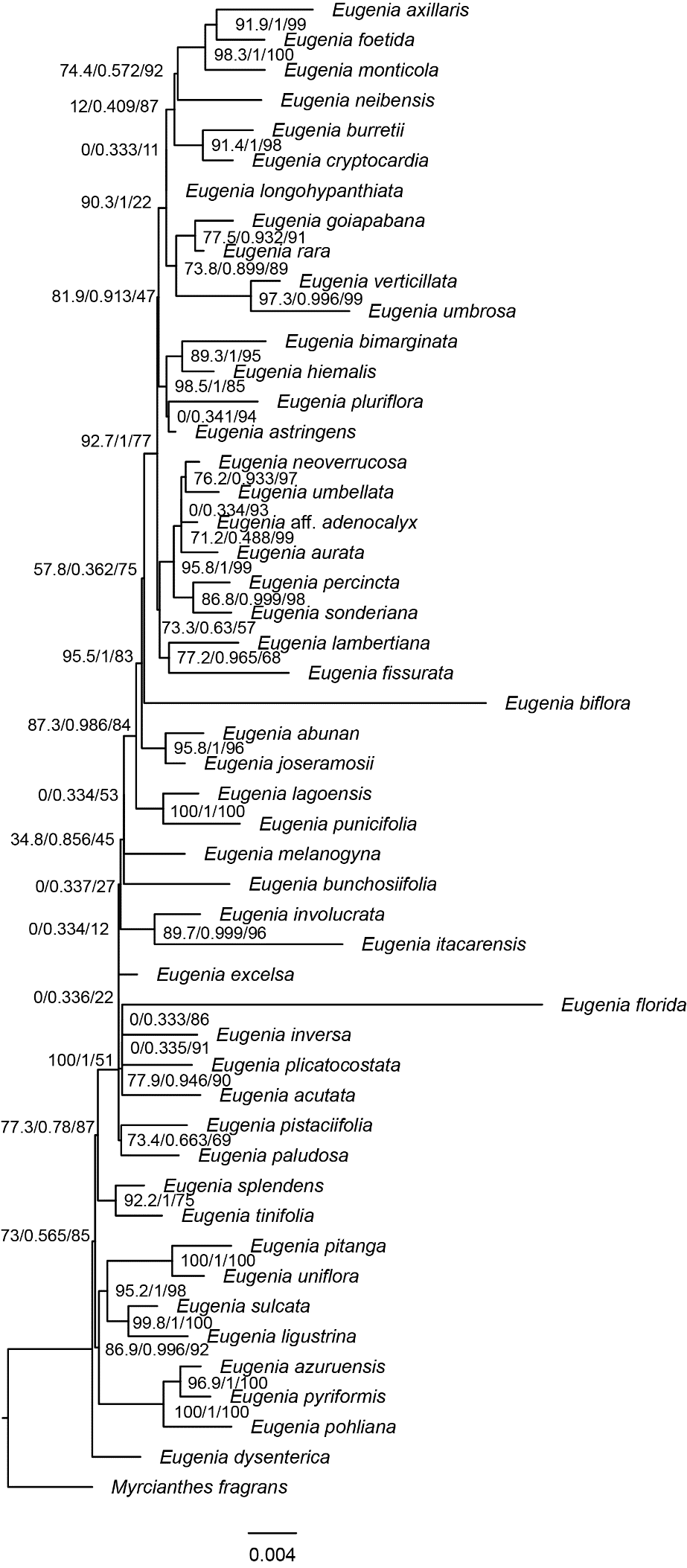


**Supplementary Figure 3.19.** Phylogenetic reconstruction of *Eugenia* based on the 44 plastome coding loci (plCDS) targeted with the Angiosperm-353 probes. Maximum Likelihood concatenated partitioned tree with bootstrap support at the nodes and additional tests to branch support with values above branches (bootstrap/aBayes /SH-aLRT /gCF/sCF). See ‘Material and Methods’ for a detailed description of the phylogenetic reconstruction. Dataset and analysis: plCDS_Cpa (See Table 1).


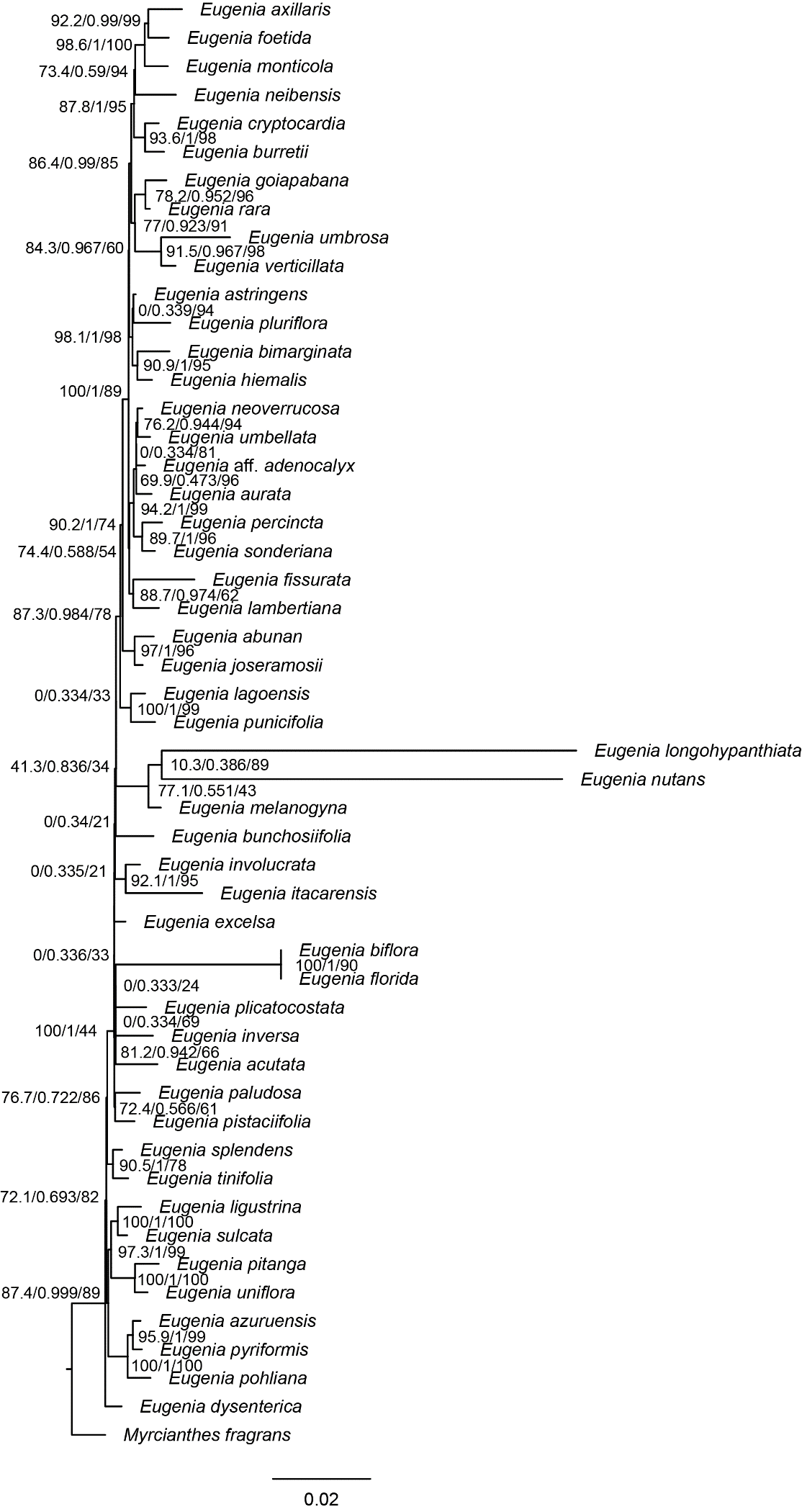


**Supplementary Figure 3.20.** Phylogenetic reconstruction of *Eugenia* based on the 44 plastome coding loci (plCDS) targeted with the Angiosperm-353 probes. Maximum Likelihood concatenated unpartitioned tree with bootstrap support at the nodes and additional tests to branch support with values above branches (bootstrap/aBayes /SH-aLRT /gCF/sCF). See ‘Material and Methods’ for a detailed description of the phylogenetic reconstruction. Dataset and analysis: plCDS_Cun (See Table 1).
