# Supplementary material for: Phylogenetic Relationships Within the Hyper-Diverse Genus Eugenia (Myrtaceae: Myrteae) Based on Target Enrichment Sequencing
Source: Front Plant Sci. 2022 Feb 4;12:759460. doi: 10.3389/fpls.2021.759460 (PMC8855041; doi:10.3389/fpls.2021.759460)
Supplement: Supplementary file 1 [file Data_Sheet_1.zip › Supplementary Table 2.DOCX]

**Supplementary Table 2.** Nuclear loci that do not fit the test of symmetry and were excluded of the phylogenetic reconstructions.

**Excluded from exons:** g4890, g5162, g5296, g5299, g5318, g5406, g5489, g5596, g5639, g5703, g5770, g5940, g5958, g6034, g6450, g6483, g6487, g6533, g6875, g6955, g7241.

**Excluded from introns**: g4954, g5038, g5064, g5138, g5162, g5220, g5260,g5271, g5318, g5343, g5347, g5421, g5449, g5454, g5463, g5489, g5531, g5551, g5594, g5599, g5614, g5744, g5791, g5816, g5821, g5822, g5843, g5849, g5859, g5894, g5913, g5933, g5941, g5945, g5960, g5974, g5990, g6000, g6004, g6034, g6036, g6051, g6114, g6119, g6128, g6139, g6164, g6175, g6198, g6295, g6303, g6320, g6389, g6398, g6412, g6439, g6460, g6498, g6527, g6528, g6531, g6639, g6641, g6649, g6667, g6705, g6738, g6782, g6797, g6825, g6848, g6854, g6859, g6860, g6883, g6913, g6933, g6946, g6947, g6961, g7021, g7136, g7241, g7279, g7313, g7331, g7363, g7602, g7628.
